# Artificial intelligence unravels interpretable malignancy grades of prostate cancer on histology images

**DOI:** 10.1038/s44303-023-00005-z

**Published:** 2024-03-06

**Authors:** Okyaz Eminaga, Fred Saad, Zhe Tian, Ulrich Wolffgang, Pierre I. Karakiewicz, Véronique Ouellet, Feryel Azzi, Tilmann Spieker, Burkhard M. Helmke, Markus Graefen, Xiaoyi Jiang, Lei Xing, Jorn H. Witt, Dominique Trudel, Sami-Ramzi Leyh-Bannurah

**Affiliations:** 1 AI Vobis, Palo Alto, CA USA; 2 Division of Urology, Department of Surgery, Centre Hospitalier de l’Université de Montréal, University of Montreal, Montréal, QC H2X 0A9 Canada; 3 Cancer Prognostics and Health Outcomes Unit, University of Montreal Health Center, Montreal, QC Canada; 4 Centre de recherche du Centre Hospitalier de l’Université de Montréal (CRCHUM), 900 Saint-Denis, Montréal, QC H2X 0A9 Canada; 5 Institut du cancer de Montréal, 900 Saint-Denis, Montréal, QC H2X 0A9 Canada; 6 University of Münster, Münster, Germany; 7 Department of Pathology and Cellular Biology, Université de Montréal, 2900 Boulevard Édouard-Montpetit, Montreal, QC H3T 1J4 Canada; 8 Department of Pathology, Centre Hospitalier de l’Université de Montréal (CHUM), 1051 Sanguinet, Montreal, QC H2X 0C1 Canada; 9 Institute of Pathology, St. Franziskus-Hospital, Muenster, Germany; 10 Department of Pathology, University of Muenster, Muenster, Germany; 11 Institute of Pathology, Elbe Klinikum Stade, Academic Teaching Hospital of The University Medical Center Hamburg-Eppendorf (UKE), Hamburg, Germany; 12 Martini-Klinik Prostate Cancer Center, University Hospital Hamburg-Eppendorf, Hamburg, Germany; 13 Department of Computer Science, University of Muenster, Muenster, Germany; 14 Department of Radiation Oncology - Radiation Physics, Stanford University School of Medicine, Stanford, CA USA; 15 Prostate Center Northwest, Department of Urology, Pediatric Urology and Uro-Oncology, St. Antonius-Hospital, Gronau, Germany

**Keywords:** Cancer imaging, Image processing

## Abstract

Malignancy grading of prostate cancer (PCa) is fundamental for risk stratification, patient counseling, and treatment decision-making. Deep learning has shown potential to improve the expert consensus for tumor grading, which relies on the Gleason score/grade grouping. However, the core problem of interobserver variability for the Gleason grading system remains unresolved. We developed a novel grading system for PCa and utilized artificial intelligence (AI) and multi-institutional international datasets from 2647 PCa patients treated with radical prostatectomy with a long follow-up of ≥10 years for biochemical recurrence and cancer-specific death. Through survival analyses, we evaluated the novel grading system and showed that AI could develop a tumor grading system with four risk groups independent from and superior to the current five grade groups. Moreover, AI could develop a scoring system that reflects the risk of castration resistant PCa in men who have experienced biochemical recurrence. Thus, AI has the potential to develop an effective grading system for PCa interpretable by human experts.

## Introduction

Prostate cancer (PCa) is one of the most prevalent malignant diseases in males and exhibits diverse cancer aggressiveness and prognosis^1^. When PCa is diagnosed, usually by biopsy, the pathological examination of cancer differentiation and dissemination status are key determinants for selecting appropriate treatments^2^. Currently, pathologists grade PCa malignancy based on the modified Gleason grading system, originally established in the 1960s^3^. The first version of the Gleason grading system was based on five tissue patterns (labeled 1–5) that identified different transformation conditions of prostatic tissues according to tissue architecture, growth, and glandular features^3,4^. This grading system produces a score that considers two identical or different patterns to grade PCa differentiation, and the order in which patterns are added differs according to tissue sampling (biopsy core vs. whole prostate)^3,4^. PCa grading was further refined after patterns 1 and 2 were mostly identified as benign with the identification of basal cells by immunohistochemistry, and some of those patterns 1 and 2 were reclassified as Gleason pattern 3 as well^5,6^. In 2016, Epstein et al. proposed a modified version of the Gleason grading system that included five grade groups (GGs) instead of nine different Gleason scores (such as 3 + 3, 4 + 3, and 5 + 3) to achieve a more concise prognostic stratification according to biochemical recurrence (BCR) rates^7^.

Despite strong prognostic capacities and continual revisions since its introduction^8^, GG reproducibility has remained limited because of interobserver variability in grading and quantification, leading to grade inconsistency even among expert pathologists, thus increasing the potential risk of treatment delay or suboptimal treatment choice^9,10^. Contemporary studies have highlighted the great potential of artificial intelligence (AI) in improving GG consistency and achieving accuracy comparable to expert levels^11–13^. However, these studies likely inherited the limitations of the current grading system as the histological ground truth is based on evaluations from a small group of expert pathologists, which is not necessarily reflective of the global pathology community (social and cognitive biases) or grading correctness^14^.

To bypass these reproducibility limitations, we applied AI to develop a novel recurrence prediction system based on long-term PCa prognosis instead of interobserver-based histology. We relied on the tissue microarray (TMA) framework of the Canadian Prostate Cancer Biomarker Network (CPCBN) initiative of the Terry Fox Research Institute; this initiative implemented thoroughly validated techniques to ensure the collection of representative samples of PCa from radical prostatectomy (RP) specimens^15^.

In this study, we developed a calibrated and interpretable algorithm for predicting PCa outcomes in multiple independent cohorts that could eventually be integrated into existing prognostic and predictive nomograms.

## Results

### Survival modeling

To establish a novel system for predicting recurrence, we initially investigated a multicenter population (CPBCN, *n* = 1489) in which the overall BCR probability was 33.1% (*n* = 493). The median time to BCR events was 26 (interquartile range [IQR], 8–52) months; in contrast, the median follow-up was 109 (76–141) months in patients without BCR events. The development and first external validation sets (CPBCN cohort) were not statistically different with respect to pathological tumor (pT) stage, pathological nodal (pN) status, and GG (Supplementary Table S1). Among 600 patients in the development set, 225 (37.5%) experienced recurrence during follow-up (median follow-up, 91 [42–123] months); in contrast, among 889 patients in the first external validation set, 268 (30.1%) had BCR (median follow-up, 75 [43–116] months).

**Fig. 1:**
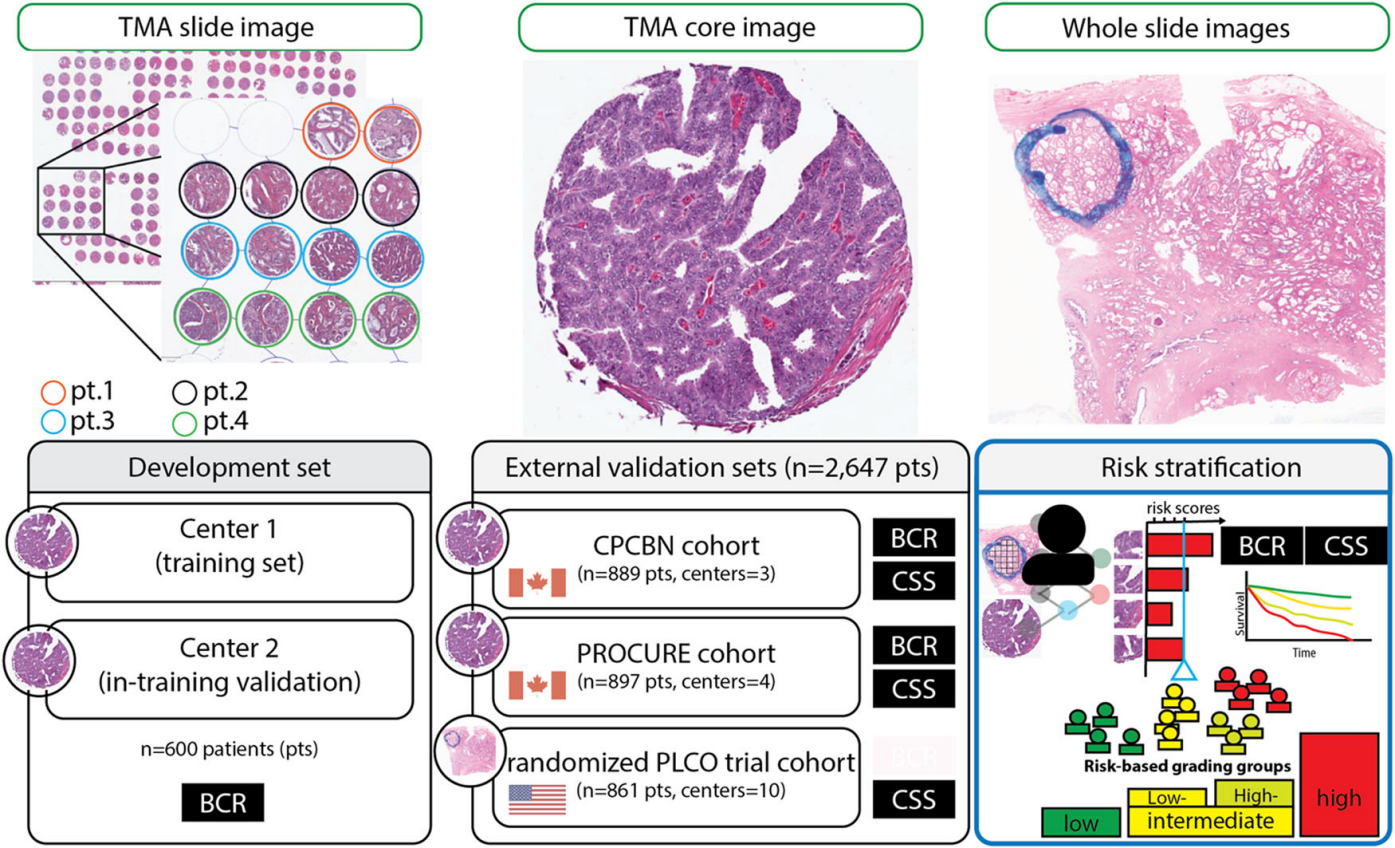
Slides from tissue microarrays (TMAs) with prostates samples from five sites were scanned, and the tissue regions were marked and extracted using QuPath (i.e., TMA slide image). We then tiled each TMA core image into patches labeled by biochemical recurrence (BCR) status to develop our BCR model. We estimated the average BCR scores for each patient and applied survival modeling to introduce our novel risk-based grading for prostate cancer. The development set consisted of 600 patients, whereas the international external validation sets included three radical prostatectomy cohorts (CPCBN, PROCURE, and PLCO). The cohort description for all datasets included in this study can be obtained from Supplementary Tables S1–S3. PLCO: The Prostate, Lung, Colorectal, and Ovarian (PLCO) Cancer Screening Trial. The endpoints we are shown in the black box. CSS: Cancer-specific survival. We emphasize that PC regions were manually demarcated on whole-slide images following the instruction given by a senior pathologist.

Figure 1 summarizes the study methodology using histology images as data input, the confidence scores for BCR as output, and the binarized recurrence status as the ground truth for model development and evaluation. The Supplementary Materials include cohort descriptions for all datasets included in this study (Supplementary Tables S1–S3).

In the first external validation set, the BCR model demonstrated a c-index of 0.682 ± 0.018 and a generalized concordance probability of 0.927 (95% CI: 0.891–0.952). The AUROC for the BCR model was 0.714 (95% CI: 0.673–0.752). Using a cutoff of 0.5 for the BCR confidence score, the sensitivity was 50.0% and the specificity was 83.2%. The precision and recall of the BCR model at a 0.5 threshold were 56.3% and 50.0%, respectively. The calibration plot demonstrated good correlation between the predicted BCR probability (BCR score) and observed 10-year BCR-free survival rate (Supplementary Figure S1).

Our novel model revealed a better effect size (hazard ratio) and higher generalized concordance probability than the classical models ResNet^16^, VGG-16^17^, and EfficientNet^18^, which were trained on the same development set for BCR prognosis. EfficientNet and the novel model provided the lowest AIC and BIC. A non-nested partial likelihood ratio test revealed that EfficientNet did not fit better than the novel model. Importantly, our novel BCR model had between 8- and 32-times fewer feature maps in the last convolutional layer for BCR prediction (before being fully connected) and a parameter capacity 125, 54-, or 24-times smaller than the models mentioned above (Supplementary Table S4). We observed no performance benefits from using image patches at ×20 or ×40 object magnifications, the attention aggregation layer, or the Cox deep convolutional model concept (Supplementary Table S5).

The results of the CHAID analysis are shown in Supplementary Figure S2. Based on the BCR scores estimated by our model and CHAID, BCR scores ≤5% were considered low risk, BCR scores between 6% and 42% were low intermediate, BCR scores between 43% and 74% were high intermediate, and BCR scores ≥75% were high risk.

### Recurrence-free survival

One study conducted univariate and multivariable Cox regression analyses on CPCBN and PROCURE cohorts to assess the prognostic value of the novel risk classification system for PCa recurrence (Supplementary Tables S6 and S7). The results showed that the BCR score was an independent prognostic factor for recurrence, along with PSA level, tumor stage, GG, and surgical margin status. The novel risk classification system showed a better model fit and superiority over GG (Table 1). No significant multicollinearity between variables was identified (VIF < 2), indicating the correlation between variables (GG and the novel risk group) is negligibly small.

**Table 1:** The model reduction and the partial likelihood ratio (LR) test revealed that a baseline model with the novel risk groups is statistically comparable to the full model to predict cancer-specific survival.

Model	Nested partial likelihood ratio test, LR (*p*-value)	AIC	BIC
*1st external validation set (CPCBN)*			
Baseline model + risk group + GG	Reference (full model)	167.5161	169.6403
**Baseline model + risk group**	**1.560 (0.213)**	**167.07**	**168.4861**
Baseline model + GG	4.114 (0.036)	169.6098	171.0259
Baseline model (pT)	7.557 (0.027)	171.0623	171.7703
*2nd external validation set (PROCURE)*			
Baseline model + risk group + GG	Reference (full model)	170.6459	174.824
**Baseline model + risk group**	**4.094 (1.2e−1)**	**172.7398**	**175.8733**
Baseline model + GG	10.056 (1.8e−3)	178.7017	181.8353
Baseline model (pT + pN)	27.777 (6.8e−5)	194.4228	196.5119

In contrast, GG (Gleason score/ISUP grade groups) was not comparable to the full model. Akaike information criterion (AIC) and Bayesian information criterion (BIC) support this finding as well since the fit of a baseline model with the novel risk groups is better than the fit of a baseline model with GG. pT: pathologic tumor stage; pN: pathologic nodal stage. +pN was excluded due to non-significance to prognose cancer-specific survival in the CPCBN external validation set. The best-performing models are highlighted in bold. Higher AIC and BIC are associated with the worst model fitness. No significant multicollinearity between variables was identified (the Variance Inflation Factors, VIF, were below 2).

**Fig. 2:**
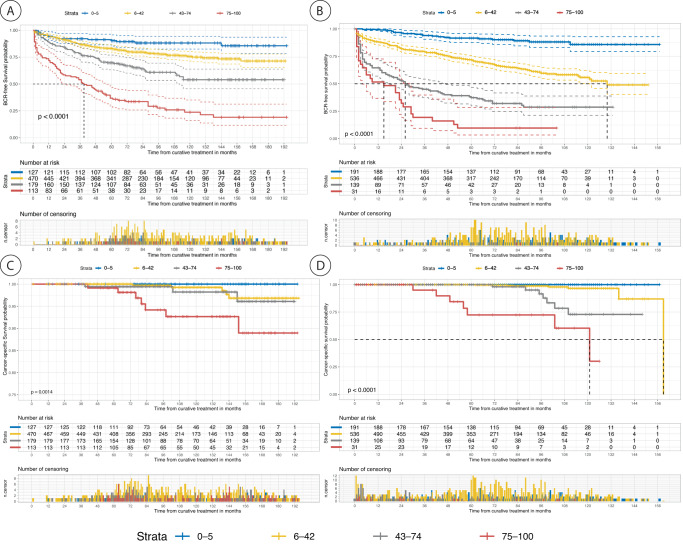

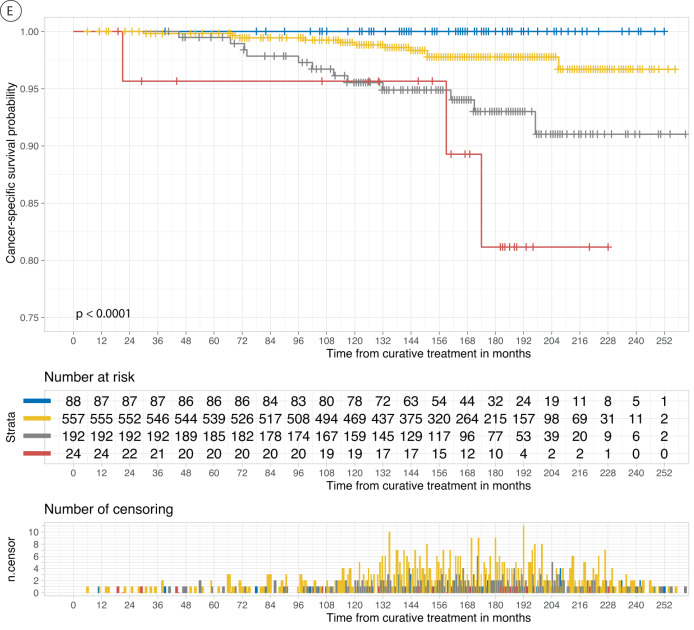
The development of Ca. **A** Kaplan–Meier curves of biochemical recurrence (BCR)-free survival according to BCR score risk stratification in the first external Validation set (CPCBN, Canada). *P*-value was measured using the log-rank test. Blue represents the low-risk group (0–5% BCR score), yellow represents the low-intermediate risk group (6–42%), gray represents the high-intermediate risk group (43–74%), and red represents the high-risk group (75–100%). The dotted lines indicate the median survival. In addition, the number of patients at risk and of censored observations are provided for the follow-up period. **B** Kaplan–Meier curves of biochemical recurrence (BCR)-free survival according to risk groups in the second external Validation set (PROCURE). Blue represents the low-risk group (0-5% BCR score), yellow represents the low-intermediate risk group (6–42%), gray represents the high-intermediate risk group (43–74%), and red represents the high-risk group (75–100%). The *p*-value was measured using the log-rank test. The number of patients at risk and of censored observations are provided for the follow-up period. **C** Kaplan–Meier curves of cancer-specific survival according to the risk groups in the first external Validation set (CPCBN, Canada). The *P*-value was measured using the log-rank test. Blue represents the low-risk group (0–5% BCR score), yellow represents the low-intermediate risk group (6–42%), gray represents the high-intermediate risk group (43–74%), and red represents the high-risk group (75–100%). The number of patients at risk and of censored observations are provided for the follow-up period. **D** Kaplan–Meier curves of cancer-specific survival according to risk groups in the second external Validation set (PROCURE, Canada). The *p*-value was measured using the log-rank test. Blue represents the low-risk group (0–5% biochemical recurrence score), yellow represents the low-intermediate risk group (6–42%), gray represents the high-intermediate risk group (43–74%), and red represents the high-risk group (75–100%). The number of patients at risk and of censored observations are provided for the follow-up period. **E** Kaplan–Meier curve of cancer-specific survival according to risk groups in the third external Validation set (PLCO, U.S.). The *p*-value was measured using the log-rank test. Blue represents the low-risk group (0–5% BCR score), yellow represents the low-intermediate risk group (6–42%), gray represents the high-intermediate risk group (43–74%), and red represents the high-risk group (75–100%). The number of patients at risk and of censored observations are provided for the follow-up period.

The survival rates varied across the novel risk groups in both the cohorts, as shown in and Fig. 2A, B (See supplementary Table S8 for 3-, 5-, 10-years BCR-free survival rates). The survival rates for GG are shown in the Supplementary section for comparison (Supplementary Tables S9 and S10 and Figures S3 and S4). The estimated power for BCR survival analysis in this study was determined to be ≥99% at an alpha level of 5% for each cohort.

### Cancer-specific Survival

This study examined cancer-specific survival using a novel risk classification system in three cohorts: the CPCBN, PROCURE, and PLCO cohorts. In the CPCBN cohort, the novel score was a significant prognostic factor for cancer-specific mortality and tumor stage; in contrast, GG was not an independent prognostic factor (Supplementary Table S11). In the PROCURE Quebec Prostate Cancer Biobank (PROCURE cohort), the novel risk score was an independent prognostic factor, along with the nodal stage; in contrast, the tumor stage was insignificant (Supplementary Table S12). Supplementary Table S13 summarizes the results of the Cox regression analyses of the PLCO cohort, further validating the independent prognostic value of the risk score for cancer-specific mortality using whole-slide images.

In the CPCBN and PROCURE cohorts, the multivariate Cox regression model with novel risk groups fit well, similar to the full model. However, the model with GG fits the data poorly (Table 2). In the PLCO cohort, both the GG and risk groups fit poorly compared with the full model, and the difference in the goodness-of-fit between the model with GG and the model with risk groups was insignificant. No significant multicollinearity between variables was identified (VIF < 2). The estimated power for BCR survival analysis in this study was determined to be ≥95% at an alpha level of 5% for each cohort. The Fine-Gray competing risk regression analyses further validated the independent prognostic value of our novel risk groups for cancer-specific mortality on external validation sets (Supplementary Tables S14–S16).

**Table 2:** The model reduction and the partial likelihood ratio (LR) test revealed that a baseline model with the novel risk groups is statistically comparable to the full model to predict cancer-specific survival.

Model	Nested partial likelihood ratio test, LR (*p*-value)	AIC	BIC
*1st external validation set (CPCBN)*			
Baseline model + risk group + GG	Reference (full model)	167.5161	169.6403
**Baseline model + risk group**	**1.560 (0.213)**	**167.07**	**168.4861**
Baseline model + GG	4.114 (0.036)	169.6098	171.0259
Baseline model (pT)	7.557 (0.027)	171.0623	171.7703
*2nd external validation set (PROCURE)*			
Baseline model + risk group + GG	Reference (full model)	170.6459	174.824
**Baseline model + risk group**	**4.094 (1.2e**−**1)**	**172.7398**	**175.8733**
Baseline model + GG	10.056 (1.8e−3)	178.7017	181.8353
Baseline model (pT + pN)	27.777 (6.8e−5)	194.4228	196.5119
*3rd external validation set (PLCO)*^a^			
Baseline model + risk group + GS	Reference (full model)	298.0581	301.8323
Baseline model + risk group	14.849 (1e−4)	310.9075	313.4237
Baseline model + GS	5.15 (0.023)	301.2158	303.732
Baseline model (prostate pathologic stage)	26.769 (1.54e−06)	320.8274	322.0855

In contrast, GG (Gleason score/ISUP grade groups) was not comparable to the full model. Akaike information criterion (AIC) and Bayesian information criterion (BIC) support this finding as well since the fit of a baseline model with the novel risk groups is better than the fit of a baseline model with GG. pT: pathologic tumor stage; pN: pathologic nodal stage. +pN was excluded due to non-significance to prognose cancer-specific survival in the CPCBN external validation set. For PLCO external validation set, we used GS provided by the study instead of GG and prostate pathologic stage (considers T, N, and M stages) due to the study history. The best-performing models are highlighted in bold. Higher AIC and BIC are associated with the worst model fitness.

^a^Since both GS and risk groups were significantly inferior than the full model, we applied the non-nested partial likelihood ratio test to compare between GS Cox model and risk group Cox model; our risk group demonstrated non-inferiority to GS, indicating comparable goodness of fit (z = 1.091, *p* = 0.138). No significant multicollinearity between variables was identified (VIF < 2).

The Kaplan–Meier curves for cancer-specific survival according to risk classification in the three external validation sets showed significant differences among the risk groups (Fig. 2C–E). Supplementary Table S17 summarizes cancer-specific survival rates across the three cohorts and shows a distinct separation of survival rates among the risk groups 10 or 15 years after RP. The low-risk group of the novel grading system had no PCa-related deaths in any of the three cohorts; in contrast, the GG in the current grading system included patients who died owing to PCa in two of the three cohorts.

PLCO cohort analysis showed that the number of slides per case and its correlation with the risk score did not significantly affect the prognostic value (Supplementary Table S18). Additional information on survival probabilities, Kaplan–Meier curves for the GG, Gleason score groups, and the PCa pathological stage is provided in Supplementary Tables S19–S21 and Supplementary Figures S5–S8 for comparison.

### Castration-resistant prostate cancer

**Fig. 3:**
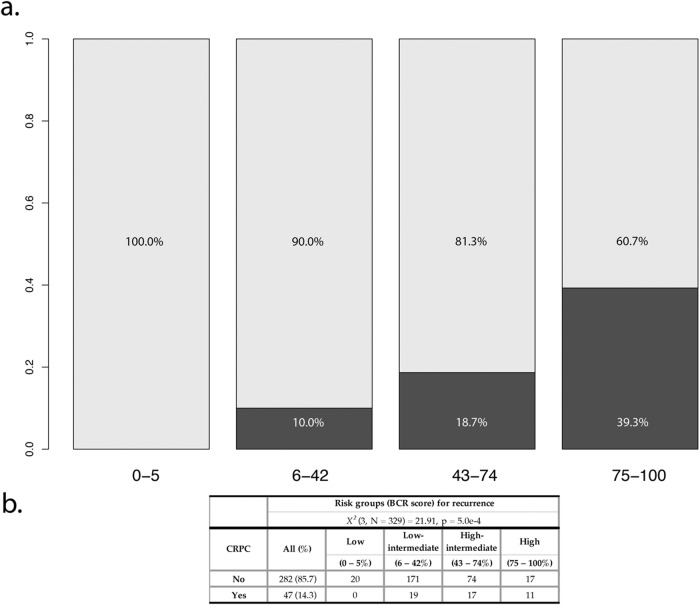
Risk Classification and CRPC Frequency in the PROCURE Cohort. Distribution of castration-resistant prostate cancer (CRPC) cases across the novel risk classification groups illustrated as bar plot (**a**) and cross tabulation (**b**) in men with biochemical recurrence (PROCURE cohort). The frequency of CRPC is significantly associated with the risk groups (Kendall’s rank correlation tau: 0.22; z = 4.2277; *p* < 0.0001).

Castration-resistant prostate cancer (CRPC) occurs when PCa progresses despite therapy-induced castrate conditions. The current study assessed the occurrence of castration-resistant prostate cancer (CRPC) in men experiencing biochemical recurrence and their association with our novel scoring and grading systems. Figure 3 shows that the proportion of CRPC increases with risk groups in men with biochemical recurrence on the PROCURE cohort. In support to this observation, we found a significant correlation between risk group and the development of CRPC (Kendall’s rank correlation tau: 0.22; z = 4.2277; *p* < 0.0001). Moreover, we identified that the low-risk group had no CRPC case and that all CRPC cases (100%) were found in the intermediate or high-risk groups. Multivariate Cox regression analysis showed that the novel risk score was an independent prognosticator for CRPC development whereas pT, pN and surgical margin status were not (Table 3).

**Table 3:** Multivariate Cox regression analysis for prognosis of castration-resistant prostate cancer (CRPC) in men with biochemical recurrence on the PROCURE external validation set.

Parameter	Hazard ratio (95% CI)	*z*	*p*	Generalized concordance probability (95% CI)
Tumor stage				
pT2	Reference			
pT3a	1.15 (0.40–3.29)	0.27	0.78863	0.536 (0.288–0.767)
pT3b	2.17 (0.81–5.78)	1.54	0.12297	0.684 (0.448–0.853)
pN1 vs. pN0/x	1.23 (0.55–2.75)	0.50	0.61392	0.552 (0.355–0.734)
PSM	1.59 (0.78–3.24)	1.26	0.20647	0.613 (0.437–0.764)
**Risk score**	**5.82 (1.72–19.73)**	**2.83**	**0.00467**	**0.853 (0.632–0.952)**

We found that the risk score is a significant and independent prognosticator for CRPC, whereas tumor and nodal stages as well as positive surgical margin status were not prognostic for CRPC (*n* = 47).

*CI* confidence interval, *PSM* positive surgical margins.

The bold value highlights the highest effect size.

### Interpretability

**Fig. 4:**
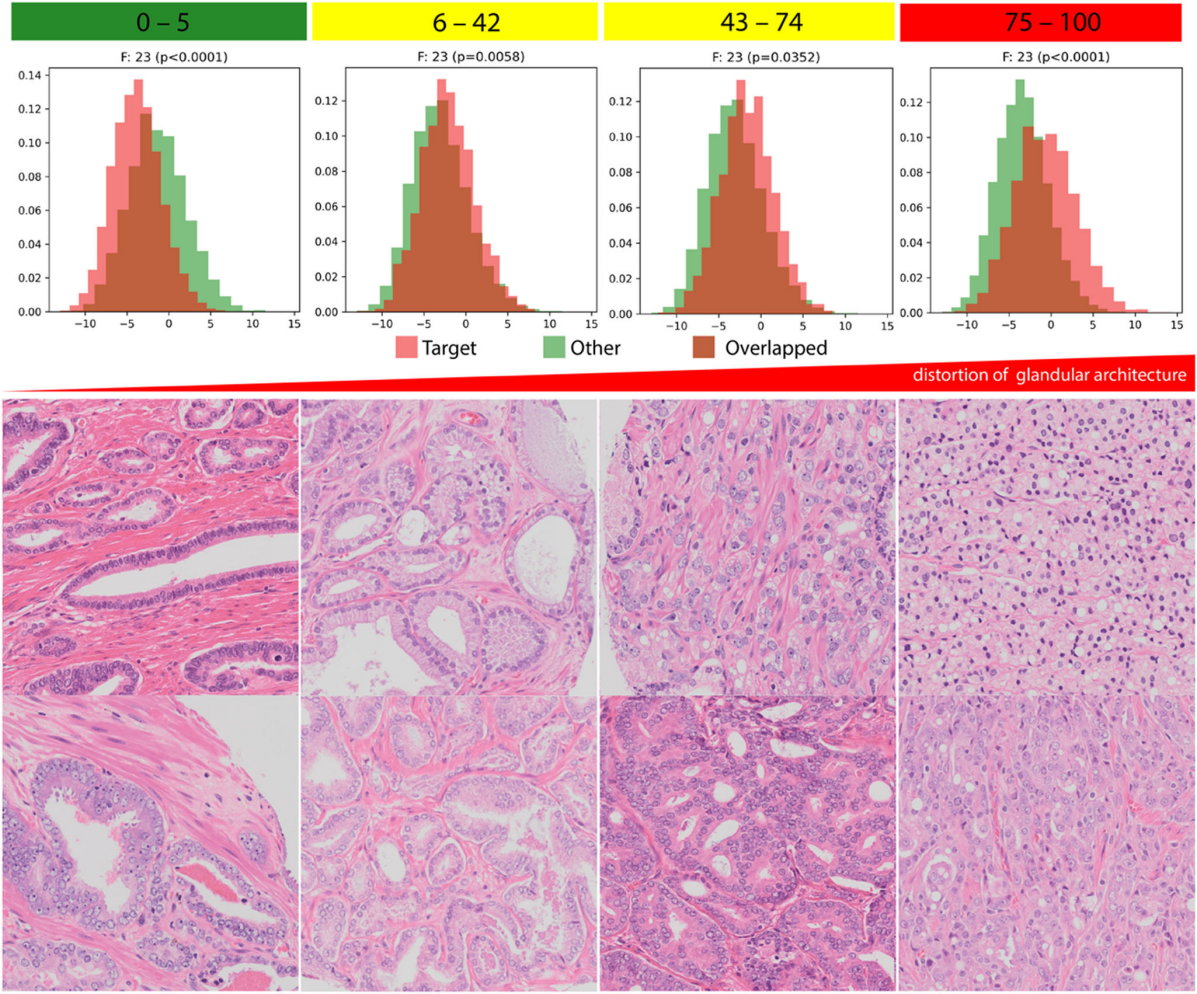
The upper row reveals the histograms for the 23rd representative feature (the 23rd feature has the highest positive weight value of 0.38 in the sigmoid prediction equation and one of the five representative features altered by BCR status) of our novel model after stratifying by the risk groups for one-vs.-other comparisons on the first external Validation set (CPCBN, Canada). The target distribution is marked in red, whereas the other distribution is marked in green; the overlapped distribution is highlighted in brown. The histology images are patches selected based on the distribution patterns (dominant red range for low and high-risk group, the overlapped range for intermediate groups). Overall, the variance for feature distributions is differed by the risk groups. Specifically, the feature distribution is shifting between these risk groups. We identified a clear histopathological gradient for distortion of glandular architecture (e.g., disappearance of organized glandular architecture) by the risk groups based on these patches. *p*-value was estimated using the Levene Test and the two-sided significance level was set to ≤0.0001. Example histology images are captured at ×10 objective magnification (~330 × 330 µm). The supplementary section includes the entire feature distribution visualization and the access information to larger image sets representing these risk groups.

Table 4 shows the concordance between the five pathologists and novel risk classifications. This table summarizes the synergistic efforts between AI and pathologists in defining a novel grading system for PCa. Despite being completely blinded to the novel risk classification and clinicopathological information, we found a striking alignment between the pathologists and risk classification in sorting image groups. Despite not relying on pattern proportions like the GG and the absent of significant collinearity between our novel risk group and GG, the image group representing the low-risk group included Gleason pattern 3 mostly; in contrast, the high-risk group included Gleason patterns 4 and 5, with Gleason pattern 3 being almost absent. The pathologists found a mixture of Gleason patterns 3 and 4 in the intermediate group, with a trend in favor of Gleason pattern 4 in the high-intermediate group. Figure 4 exemplary illustrates the histopathological gradient for distortion of glandular architecture as well as the Supplementary section include information on accessing image groups.

**Table 4:** Image group assessment by pathologist in accordance with the BCR score risk stratification with decision explanation.

Pathologist	Sorting agreement with AI	Explanation
Pathologist A	Yes	Group A: Dominant Gleason pattern 3 Group B: Mixed Gleason pattern 3 and 4, cannot determine which pattern is dominant. Group C: Mixed Gleason pattern 3 and 4, but Gleason pattern 4 is dominant. Group D: Few Gleason pattern 3, mostly Gleason pattern 4 and/or 5.
Pathologist B	Yes	Group A has the most favorable malignancy grade (mostly Gleason pattern 3), whereas Group D has the worst differentiation grade (hard to identify Gleason pattern 3, mostly Gleason pattern 4 and 5). Cannot determine a significant difference in pattern distribution in Group B and C, but it seems to me that C has more Gleason pattern 4.
Pathologist C	Yes	There is a pattern trend in these image groups, likely driven by Gleason pattern 3. For example, Group A has mostly Gleason pattern 3 and Group D has mostly Gleason pattern 4 and 5. This trend is, however, not clearly visible in group B and C (mixed Gleason patterns 3 and 4).
Pathologist D	Partially	Group A: Dominantly favorable Gleason pattern 3 Group B: Mixed Gleason patterns 3 and 4 Group D: Dominantly Gleason pattern 4 and 5 (Mostly Gleason score at least 8) Group C: Mostly heterogenous prostate cancer constellation with dominance of Gleason pattern 4.
Pathologist E	Yes	Group A: Dominant Gleason pattern 3. Rare occurrences of mucinous cribriform, glomeruloid patterns Group B: Mixed Gleason pattern 3 and 4, with rare occurrence of single cells (pattern 5). When pattern 4 is present, it is mostly ill-defined, sometimes cribriform. Group C: Dominant pattern 4, with rare occurrences of pure pattern 3. Pattern 4 is often cribriform. Group D: Dominant patterns 4 and 5.

The image groups, which were randomly assigned, required sorting by the pathologists. Pathologist D weighted the heterogeneity definition to rank group D and C, thereby resulting into the partial agreement.

The in-depth evaluation of 64 representative features revealed that specifically the 23rd representative feature showed two distinct distributions (different variances) according to the risk groups and the recurrence status (Levene test, *P* < 0.0001). According to the histogram and bimodal (one-vs-other) distribution comparisons, the feature distribution for low or high-risk group was noticeably more distinguishable than the feature distribution for low- or high-intermediate groups (Fig. 4). The evaluation of image patches selected according to the feature distribution (dominant red range for low and high-risk group, the overlapped range for intermediate groups) revealed a histopathology pattern gradient across the risk groups (Fig. 4). Supplementary Figures S9–S13 provide the distribution patterns for 64 feature representations stratified by recurrence status and risk groups.

Gleason pattern 5 was mostly observed in the lower intermediate risk group (31% for CPCBN and 27% for PROCURE), higher intermediate/high-risk groups (67% for CPCBN and 73% for PROCURE). GG2 (3 + 4) predominantly belonged to intermediate risk groups, accounting for 76% in PROCURE and 80% in CPCBN. Within these intermediate risk groups, GG2 was predominantly found in the lower intermediate risk group, making up 76% in CPCBN and 88% in PROCURE.

## Discussion

In this study, we developed and externally validated a novel grading system for PCa that was superior to the existing grading systems. We demonstrated that AI could be a helpful tool for generating a well-calibrated grading system interpretable by human experts, including risk stratification groups with distinct survival probabilities that enable communication with and between domain experts and between patients and experts to make clinical decisions^7,19,20^. A well-calibrated deep learning model significantly mitigates the usual concerns of overconfidence and enables the interpretation of the model’s prediction as scores^21,22^. Lastly, risk stratification further enables the exploration of common histopathologic patterns by risk scores^7,19,20^.

Previous AI efforts have focused on replicating grading systems using supervised learning. Bulten et al. reported a deep learning model trained with the semi-automatic region-level annotation technique and slide-level annotations to show a Cohen’s quadratic kappa score (κ_quad_) of 0.918 (95% CI 0.891–0.941)^11^. Similarly, Ström et al. developed an ensemble of deep learning models trained with automatically generated region-level annotations from pen marks and slide-level annotations, yielding a linear-weighted kappa score (κ_lin_) of 0.83^23^.

A recent study proposed a weakly supervised deep learning model that leveraged only the global Gleason score of whole-slide images during training to grade patch-pixel-level patterns and perform slide-level scoring accurately^24^. The authors reported an average improvement on Cohen’s quadratic kappa score (κ_quad_) of approximately 18% compared to full supervision for the patch-level Gleason grading task^24^. Similarly, another study reported that the use of the AI-assisted method was associated with significant improvements in the concordance of PCa grading and quantification between pathologists: pathologists 1 and 2 had 90.1% agreement using the AI-assisted method vs. 84.0% agreement using the manual method (*p* < 0.001)^25^.

Despite these results being promising, the current grading system still suffers from reader dependency, and any AI-based solution developed to improve the interrater agreement for tumor grading will apply to a closed network of human readers with associated social and cognitive biases. To address these integral notions of AI design, our grading system was calibrated with different risk groups independent of human readers. Our approach also overcomes the challenges of interpreting an AI-designed grading system as human readers can identify pattern trends in our grading system. Finally, our novel grading system accurately facilitated PCa grading at the clinically relevant case level using a limited number of representative PCa tissues (three to four small regions representing the index PCa on an RP specimen) or a fully representative slide from an RP specimen.

Previous studies have explored the potential of digital biomarkers or AI-based Gleason grading systems for survival prediction and prognosis in PCa. For instance, a most recent nested case-control study developed a prognostic biomarker for BCR using ResNet-50D^26^ and a TMA cohort, and the time to recurrence was utilized to label the histology images^27^. Wulczyn et al. proposed an AI-based Gleason grading system for PCa-specific mortality based on Inception^12^-derived architecture^28^. Yamamoto et al. utilized deep autoencoders^29^ to extract key features that were then fed into a second machine learning model (regression and support vector machine^30^) to predict the BCR status for PCa at fixed follow-up time points (Year 1 and 5)^31^. Other studies also utilized multimodal data (molecular feature and histology) for prognosis in different cancers^32,33^. Overall, these studies set the ground for further survival analyses using AI; however, they were limited by the post hoc explanation of their black box models that is not necessarily reflective of interpretable, clinically relevant well-validated algorithms^34–36^.

Our novel grading system is also prognostic for the development of CRPC which represents an advanced progression stage of PCa with poor outcome, that no longer completely responds to the androgen deprivation therapy and consequently continues to progress^37,38^. Our data demonstrate the potential use of our novel grading system as clinical tool to determine cases at high-risk of CRPC development and accordingly propose a risk-adapted personalized surveillance strategy.

One of the most important aspects to consider when developing tools for clinical decision-making is practicality and clinical utility. Our novel model was calibrated to predict 10-year BCR-free survival probability and facilitate model interpretation. It should also be noted that the standard prognostic factors for PCa are all obtained during diagnosis or treatment without accounting for any time information. Accordingly, we integrated this important aspect into our novel prediction system and selected model architectures for comparison based on recent surveys for medical imaging^39,40^ and the PANDA Challenge^41^ for PCa. Similarly, because c-index and ROC curves are not ideal for comparing prognostic models, we utilized the partial LR test, AIC, and BIC to identify which model configuration fits better and provides a superior prognostic performance^42^. The novel prediction system presented in this study does not rely on Cox models to calculate risk scores and determine risk groups. In this study, Cox models were used only to evaluate the accuracy and clinical utility of the grading system.

This study applied the Gleason grading system for nomology and ontology to describe the histopathological contents of each group as it is widely accepted as a communication terminology for histopathological changes in PCa among domain experts (including urologists, pathologists, and oncologists), despite their interrater limitations. Although there was some unsurprising overlap between our risk scores and the GG, the risk groups provided significantly different interpretations of the GG patterns. Furthermore, our analysis revealed no significant evidence of multicollinearity among various parameters, including Gleason grade (GG) and the risk groups. This suggests that the variables we considered in our study are independent and not significantly correlated with each other.

We limited the sampling dimension to 0.6 mm (utilizing TMA cores) while evaluating the interpretability of our novel grading system. This restriction enabled us to improve the readability of the histological content associated with the risk score. Our TMA cohorts were assembled through a meticulous process involving rigorous protocols and quality control components to ensure the sampling of representative prostate cancer tissues for each respective case^15,43^.

We adopted the definition proposed by Rudin for interpretable AI^34^, which obeys a domain-specific set of constraints so that human experts can better understand it. Interpretable AI necessitates a calibrated model, a requirement that aligns with its importance in clinical decision-making, whereas post hoc explanation of a black box model does not necessarily equate to interpretable AI^34,44^. Moreover, within the domain of deep neural networks, the model generalization primarily arises from the presence of a substantial inductive bias intrinsic to their architectural design; notably, deep neural networks demonstrate behavior that closely approximates Bayesian principles, as substantiated by prior research^45–48^. This specific property strengthens our assumption that bimodal distributions linked to the corresponding risk groups are observable for certain features within the penultimate fully connected layer, as demonstrated in Fig. 4 and Supplementary Figures S9 to S13; such alteration in the bimodal distribution across different risk groups provides insights into the model’s inference and the feature distributions resulting from the input images.

Although our results are robust, and our novel grading system does not rely on GG nor pattern proportions, whether it can overcome sampling errors, tissue fragmentation, degradation, or artifacts caused by prostate biopsy and/or poor RP tissue quality is unknown. We did not evaluate our grading system on the biopsy materials for survival modeling as a sampling effect (evident from the increase in PCa on RP) and the effects of time or intermediate events (such as cancer progression) until treatment (such as RP) were difficult to control in the experimental setting. In contrast, these effects were easier to control with RP specimens, and it was previously demonstrated that TMA, corresponding biopsy samples and RP specimens were comparable to GG^15,43^. The selection strategy for whole-slide images (WSIs) or tissue microarray (TMA) sampling in the current cohorts was determined exclusively by the study organizers before the initiation of the current study. Thus, our strategy mitigated the observer bias by ensuring that data collectors were not involved with data analysis process of the current study. Although we did not have control over the WSI or TMA sampling and case selection process for the current study, our power analyses indicate that the sample size we have is adequate to execute our study. Moreover, the TMA cohorts were primarily designed for biomarker validation, specifically to assess the effectiveness of biomarkers in predicting or prognosing survival outcomes. The selection of TMA samples accordingly followed predetermined criteria set by the study organizers to ensure accurate representation and robust validation while mitigating the selection bias^15,43^. To mitigate potential bias from interobserver variability in labeling histopathological image groups, we requested explanations from pathologists to better understand the factors influencing their decisions. This approach aimed to improve transparency and provide insights into the potential sources of bias in the interpretation of histopathological images. Finally, our AI-based grading system was not developed to detect PCa; therefore, additional models to detect PCa are required for a fully automated grading system.

This study introduced and validated a novel grading system resulting from the synergy between AI and domain knowledge. Future research should focus on identifying the application boundaries of our novel grading system in a real-world setting, including its possible integration into existing nomograms used to predict prognosis and treatment response.

## Methods

### Data

#### Cohorts

In this study, we adopted a study design that focused on the analysis of independent retrospective cohorts. The patients with CPCBN were randomly divided into development and validation sets based on their institutions. The development cohort included 600 RP cases from two institutions in the CPCBN framework^15,43^. Each center received ethical approval from their Institutional Review Board (IRB) for biobanking activity and for their contributions to the CPCBN. CTRNet standards were followed for quality assurance and ensured appropriate handling of human tissue^49^. The first external validation set, the CPCBN cohort, included 889 RP cases from three different institutions within the CPCBN framework, anonymized to minimize bias and excluding the institutions used in the development set to avoid potential label leakage. The second cohort included 16 digital TMA scans of 897 patients from the PROCURE cohort^50,51^. The study has been approved by the McGill University Institutional Review Board (study number A01-M04-06A). Lastly, the 1502 H&E-stained whole-slide images from 861 RP cases in the Prostate, Lung, Colorectal, and Ovarian (PLCO) Cancer Screening Trial (NCT00339495; PLCO cohort) were used^52,53^. Only cores or representative slides from the RP index lesion were used to develop and validate the malignancy grading system for PCa. Access to the PLCO data set was approved through the National Cancer Institute Cancer Data Access System. Informed consent was obtained from all subjects involved in the study and managed by the respective organizers. The current study was conducted in accordance with the Declaration of Helsinki, and the respective study organizers were responsible for obtaining the ethical approval. The Supplementary Methods details TMA construction and histological images of these cohorts as well as their exclusion and inclusion criteria.

#### Clinicopathological information

Histological images of PCa, clinicopathological information, and longitudinal follow-up data were available for all cases. Clinicopathological data included age at diagnosis, preoperative prostate-specific antigen (PSA) measurements, RP TNM classification, and RP GG for all patients at the RP and TMA core sample levels. Tumor staging was based on the 2002 TNM classification^54^ and grading according to the 2016 WHO/ISUP consensus^55^. All data were available from the corresponding framework and study trial. The clinicopathological information was obtained through a meticulous chart review process, involving the extraction of data and the data quality control from the electronic health records (EHR) of each participating hospital.

#### Follow-up and endpoints

Most patients were regularly followed after RP to identify BCR, defined as two consecutive increases in serum PSA levels above 0.2 ng/mL, PSA persistence (failure to fall below 0.1 ng/mL), initiation of salvage or adjuvant treatment, and cancer-specific death. BCR status (non-BCR vs. BCR) and cancer-specific death status were documented during the follow-up period. Non-BCR cases or cancer survivors with incomplete follow-up duration were censored at the date of last follow-up for survival analyses. The occurrence of the castration-resistant prostate cancer (CRPC) during the follow-up period was additionally documented.

### Model development

The development cohort was further divided into training and in-training validation sets, with the largest single-institution cohort used as the training set. Gleason patterns were utilized to ensure consistent histological appearance in circular cores with a diameter of approximately 0.6 mm. Gleason patterns 3 + 3 and 4 + 4 were specifically used to evaluate homogeneous cores to ensure consistency in the histological appearance. These patterns were selected to determine the minimum and maximum ranges of architectural tissue alteration within the circular cores. In contrast, cores with Gleason pattern 4 + 3 were considered to represent heterogeneous cores, indicating an intermediate stage of architectural alteration of the tissue. The selection of Gleason pattern 3 cores was limited to cases without recurrence during follow-up to ensure a clean pattern. Images including Gleason pattern 5 were intentionally excluded from the training set. By removing pattern 5 and 3 + 4 from the training set, we aimed to encourage the model to learn and rely on other distinguishing features that are indicative of different malignancy patterns other than the Gleason pattern system (quasi zero-shot learning). As a result, the model development process accounted for tissue appearance and distortion variations independent of the current Gleason grading system.

The study employed neural architecture search using PlexusNET and grid search to find the best architecture model for BCR prediction^56^. ADAM optimization algorithm and cross-entropy loss function were used to train the models^57^. The optimal architecture was selected based on a 3-fold cross-validation performance. The resulting model was trained on the entire training set with early stopping and triangular cyclical learning rates applied to mitigate overfitting. Model performance was evaluated at the case level using confidence scores and metrics such as AUROC and Heagerty’s c-index^58–60^. Tile-level predictions were aggregated to determine core- or slide-level predictions, and case-level predictions were estimated by averaging core- or slide-level predictions.

In parallel, we repeated the same steps using ResNet-50RS^16,61^, VGG-16^17^, and EfficientNet^18^, as these represent state-of-the-art or classical architectures (SOTA)^16–18,61^, and we then assessed the effect sizes (i.e., hazard ratio) for each model for BCR prognosis at case level. In a similar manner, we tested the performance benefits of using image patches at ×20 or ×40 object magnification, using the COX deep convolutional neural network concept as described by Katzman et al.^62^ or the attention aggregation layer^63^ instead of the global average pooling for our survival modeling.

The risk classification model for BCR was constructed using the chi-square automatic interaction detector (CHAID) algorithm^64^, with probabilities cutoffs identified on the development set and validated on external validation sets.

### Model evaluation

In the development and external validation cohorts, confidence scores for BCR (BCR scores) were generated for all cases. Prognostic classification and accuracy were measured using AUROC, Heagerty’s C-index, and generalized concordance probability. The goodness-of-fit was assessed using Akaike information criterion (AIC) and Bayesian information criterion (BIC)^65–67^.

Calibration plots were created for external validation of the BCR model to evaluate its interpretability. Harrell’s “resampling model calibration” algorithm was applied to assess model calibration^68,69^. BCR predictions were compared to corresponding Kaplan–Meier survival estimates within 10 years.

Univariate and multivariate weighted Cox regression analyses were conducted on external validation cohorts using Schemper et al.’s method to provide unbiased hazard ratio estimates, even in cases of non-proportional hazards^70^. Parameters included age at diagnosis, surgical margin status, preoperative serum levels of PSA, pT stage, pN stage, GG, and BCR confidence scores. Parameters significant in the univariate analysis were included in the multivariate Cox regression analysis to identify independent prognostic factors for BCR.

Cox regression models were used for cancer-specific survival to examine the prognostic value of the novel score/grading system, including GG, tumor stage, and the novel score/grading system. In addition to that, we performed the Fine-Gray competing risk regression analyses for cancer-specific mortality, while considering other competing causes of death reported in the death certificates. Kaplan–Meier survival estimates were used to approximate the BCR and cancer-specific survival probabilities for GG and the novel risk classification.

Nested partial likelihood ratio tests were conducted to compare different Cox regression model configurations (only categorical variables) and determine the best model for prognosis^71^. The best-performing grading system (novel grading vs. GG) was chosen based on the lowest changes in partial likelihood ratio and *p*-values. The AIC and BIC values were compared among the Cox regression models, with the best model having the lowest values. Pearson correlation coefficient was calculated to assess the correlation between the risk score and slide number.

The variance inflation factor (VIF) was utilized to assess the multicollinearity level between the GG, novel grading, and tumor stage. Here, we built two logistic regression models for 10-year BCR and cancer-specific death prediction. VIF below 2 indicates a negligible multicollinearity between these prediction variables.

To ensure the robustness, reliability, and adequate sample size of our study, we conducted a power calculation for Cox proportional hazards regression. Specifically, we evaluated the statistical power of our analysis considering GG and risk score groups to prognose BCR or cancer-specific mortality using powerSurvEpi^72^.

### Human interpretability

The first external validation set (CPCBN) core images were grouped according to their risk classification. Five experienced genitourinary pathologists with over 10 years of expertise were asked to review and sort randomly labeled image groups based on tumor differentiation. Furthermore, these senior pathologists had to explain their decision in sorting the image groups while no specific instruction on how to explain their decision was given. Pathologists were blinded to the corresponding clinicopathological and follow-up information to mitigate the recall bias and survivorship bias. Each pathologist was individually approached via email to perform the assigned task while the image groups were randomly sorted before sharing them with each pathologist; no communication between pathologists specific to this task was permitted to avoid the confirmation bias. Time limitation was not set to execute the task. To assess the inter-rater agreement between a pathologist and our novel risk groups, we utilized a percent agreement based on the proportion of correctly labeled risk groups out of the total number of risk groups under the assumption that the probability for a random agreement in sorting the entire grouped images between a single pathologist and the novel risk classification model is <5% and therefore negligible.

### Software

Model development and analyses were performed with Albumentations^73^, Keras 2.6^74^, TensorFlow 2.6^75^, Python™ 3.8, SPSS® 23, and the R statistical package system (R Foundation for Statistical Computing, Vienna, Austria).

## Supplementary information

Supplementary Information

## Data Availability

Due to data transfer agreements and data privacy issues, data cannot be made openly available.

## Code availability

An abstract version of the codes can be obtained from https://github.com/oeminaga/AI_PCA_GRADE.
