# Author Correction: Artificial intelligence unravels interpretable malignancy grades of prostate cancer on histology images

**DOI:** 10.1038/s44303-024-00026-2

**Published:** 2024-07-08

**Authors:** Okyaz Eminaga, Fred Saad, Zhe Tian, Ulrich Wolffgang, Pierre I. Karakiewicz, Véronique Ouellet, Feryel Azzi, Tilmann Spieker, Burkhard M. Helmke, Markus Graefen, Xiaoyi Jiang, Lei Xing, Jorn H. Witt, Dominique Trudel, Sami-Ramzi Leyh-Bannurah

**Affiliations:** 1 AI Vobis, Palo Alto, CA USA; 2 Division of Urology, Department of Surgery, Centre Hospitalier de l’Université de Montréal, University of Montreal, Montréal, QC H2X 0A9 Canada; 3 Cancer Prognostics and Health Outcomes Unit, University of Montreal Health Center, Montreal, QC Canada; 4 Centre de recherche du Centre Hospitalier de l’Université de Montréal (CRCHUM), 900 Saint-Denis, Montréal, QC H2X 0A9 Canada; 5 Institut du cancer de Montréal, 900 Saint-Denis, Montréal, QC H2X 0A9 Canada; 6 University of Münster, Münster, Germany; 7 Department of Pathology and Cellular Biology, Université de Montréal, 2900 Boulevard Édouard-Montpetit, Montreal, QC H3T 1J4 Canada; 8 Department of Pathology, Centre Hospitalier de l’Université de Montréal (CHUM), 1051 Sanguinet, Montreal, QC H2X 0C1 Canada; 9 Institute of Pathology, St. Franziskus-Hospital, Muenster, Germany; 10 Department of Pathology, University of Muenster, Muenster, Germany; 11 Institute of Pathology, Elbe Klinikum Stade, Academic Teaching Hospital of The University Medical Center Hamburg-Eppendorf (UKE), Hamburg, Germany; 12 Martini-Klinik Prostate Cancer Center, University Hospital Hamburg-Eppendorf, Hamburg, Germany; 13 Department of Computer Science, University of Muenster, Muenster, Germany; 14 Department of Radiation Oncology - Radiation Physics, Stanford University School of Medicine, Stanford, CA USA; 15 Prostate Center Northwest, Department of Urology, Pediatric Urology and Uro-Oncology, St. Antonius-Hospital, Gronau, Germany; 16 University Medical Centre Mannheim, Universitätsklinikum Mannheim, Theodor-Kutzer-Ufer 1-3, Mannheim, 68167 Germany

**Keywords:** Cancer imaging, Image processing

Correction to: *npj Imaging* (2024) 10.1038/s44303-023-00005-z, published online 06 March 2024

In the author’s contribution, the following statement was missing ‘TS evaluated the histological images.’. In the figure caption 2 the title “Kaplan-Meier Survival Analyses.” was missing and for the author “Jorn H. Witt” following affiliation was missing “University Medical Centre Mannheim, Universitätsklinikum Mannheim, Theodor-Kutzer-Ufer 1-3, 68167 Mannheim, Germany”.

