# Supplementary material for: Artificial intelligence unravels interpretable malignancy grades of prostate cancer on histology images
Source: Npj Imaging. 2024 Mar 6;2:6. doi: 10.1038/s44303-023-00005-z (PMC12118760; doi:10.1038/s44303-023-00005-z)
Supplement: Supplementary file 1 — Supplementary Information [file 44303_2023_5_MOESM1_ESM.pdf]

Supplementary Material "Artificial Intelligence Unravels Interpretable Malignancy Grades of Prostate Cancer on Histology Images"

Table of Contents

COHORT DESCRIPTION 3

THE IMAGE COHORT OF THE CANADIAN PROSTATE CANCER BIOMARKER NETWORK'S FRAMEWORK 3
THE IMAGE COHORT OF QUEBEC PROSTATE CANCER BIOBANK (PROCURE) 3
THE IMAGE COHORT OF PROSTATE, LUNG, COLORECTAL, AND OVARIAN (PLCO) CANCER SCREENING TRIAL 4

IMAGE EXTRACTION FROM TISSUE MICROARRAY (TMA) SLIDE IMAGES 4

IMAGE PREPROCESSING 4

SUPPLEMENTARY TABLE S1 5

SUPPLEMENTARY TABLE S2 6

SUPPLEMENTARY TABLE S3 7

SUPPLEMENTARY TABLE S4 8

SUPPLEMENTARY TABLE S5 9

SUPPLEMENTARY TABLE S6 10

SUPPLEMENTARY TABLE S7 11

SUPPLEMENTARY TABLE S8 12

SUPPLEMENTARY TABLE S9 13

SUPPLEMENTARY TABLE S10 14

SUPPLEMENTARY TABLE S11 15

SUPPLEMENTARY TABLE S12 16

SUPPLEMENTARY TABLE S13 17

SUPPLEMENTARY TABLE S14 18

SUPPLEMENTARY TABLE S15 19

SUPPLEMENTARY TABLE S16 20

SUPPLEMENTARY TABLE S17 21

SUPPLEMENTARY TABLE S18 22

SUPPLEMENTARY TABLE S19 23

SUPPLEMENTARY TABLE S20 24

SUPPLEMENTARY TABLE S21 25

SUPPLEMENTARY FIGURE S1 26

SUPPLEMENTARY FIGURE S2 27

SUPPLEMENTARY FIGURE S3 28

SUPPLEMENTARY FIGURE S4 29

SUPPLEMENTARY FIGURE S5 30

SUPPLEMENTARY FIGURE S6 31

SUPPLEMENTARY FIGURE S7 32

SUPPLEMENTARY FIGURE S8 33

REFERENCES 39

Cohort description

The image cohort of the Canadian Prostate Cancer Biomarker Network's Framework

Five different biobanks affiliated with Canadian academic health care centers contributed radical prostatectomy (RP) specimens from 1990 and 2011 to the Canadian Prostate Cancer Biomarker Network (**CPCBN**): Centre hospitalier de l'Université de Montréal (CHUM), Center de recherche du CHU de Québec-Université Laval (CHU de Québec-UL), McGill University Health Centre (MUHC), Vancouver Prostate Centre (VPC), and the University Health Network (UHN). All subjects signed informed consent to contribute to one of the participating biobanks, and each institution's ethical board approved this study, and institutional authorization was obtained.[1,2] RP as primary treatment was performed within six months after the primary diagnostic biopsy. The CPCBN biorepository is complemented by data on diagnosis, treatment, and long-term clinical outcomes. Tumor and lymph node staging was performed according to the 2002 TNM classification by the AJCC/UICC (American Joint Committee on Cancer/International Union Against Cancer).[3] Tumor grading was evaluated according to the 2016 WHO/ISUP consensus.[4] Only cases without neoadjuvant androgen-deprivation therapy were considered for CPCBN.

TMAs were constructed and designed from the RP specimens from 1,489 patients using a comprehensive protocol with rigorous tissue quality assessment, including immunohistochemistry staining, independent double central pathology review by genitourinary expert pathologists as described previously, and routine histology slides superimposed against immunohistochemistry targeting basal cells.[1,2] Each TMA block contained three to four cores from the RP index lesion and one to two cores from adjacent benign tissues within the same individual. Our study considered only cores from the RP index lesion to develop a malignancy grading system for prostate cancer (PCa).

The image cohort of Quebec Prostate Cancer Biobank (PROCURE)

A total of 16 digital TMA scans representing 897 cases from the prospectively maintained **PROCURE** Quebec Prostate Cancer Biobank were utilized for external validation.[5,6] The biobank includes patients with localized PCa, who were treated with RP between November 2006 and April 2013 in one of the following University hospitals: Laval University, McGill University, Université de Montréal, and Université de Sherbrooke (**Supplementary Table S2**). Only cases without neoadjuvant androgen-deprivation therapy were enrolled. Our cases were selected to curate a representative cohort with a minimum median follow-up of 5 years and sufficient image quality, i.e., no tissue degradation/fragmentation or tissue damages and ≥50% tumor burden per TMA spot. All TMA slides of the PROCURE cohort were stained with H&E and digitally scanned at 20x objective magnification using a Leica Biosystems device (Wetzlar, Germany) and stored in SVS format. We then utilized QuPath[7] (Dublin, Ireland) to extract core images (1450 × 1450 pixels or 717.75 × 717.75 µm where 0.4950 µm corresponds to one pixel) as described above. Histology images representing PCa and corresponding clinicopathological information and longitudinal follow-up data were available for all cases. Clinicopathological data included age at diagnosis, preoperative prostate-specific antigen (PSA) measurement (ng/mL), RP TNM classification, and RP GGs.

The image cohort of Prostate, Lung, Colorectal, and Ovarian (PLCO) Cancer Screening Trial

A third external validation set consisted of 1,502 H&E-stained whole slide images from 861 RP cases enrolled in the Prostate, Lung, Colorectal, and Ovarian (PLCO) Cancer Screening Trial (NCT00339495).[8,9] This study used a linkage with the National Death Index to extend mortality follow-up to a maximum of 19 years after randomization.[10] Only cases without neoadjuvant androgen-deprivation therapy were selected. The slides of the RP **PLCO cohort** were obtained from 10 US centers and digitally scanned at 40× objective magnification (one pixel corresponds to 0.2532 μm) using a Leica Biosystems device (Wetzlar, Germany) and stored in SVS format. The cancer lesions were marked on the slides by PLCO pathologists, and a single PCa researcher of our team digitally verified and delineated these lesions using Aperio Image Scope (Leica Biosystems, Wetzlar, Germany) at 10x magnification, which was then verified by two pathologists. In addition, clinicopathological information was available at the case level.

Histology images representing PCa and corresponding clinicopathological information and longitudinal follow-up data were available for all cases. Clinicopathological data included age at diagnosis, preoperative prostate-specific antigen (PSA) measurement (ng/mL), RP TNM classification, and RP grade groups (GGs) for all PCa patients at RP and at TMA core sample level if applicable.

Image extraction from tissue microarray (TMA) slide images

All TMA slides were stained with hematoxylin and eosin (H&E) and digitally scanned at 40× objective magnification using an Olympus scanner device (Tokyo, Japan) and stored in TIFF format. We then utilized QuPath[7] (Dublin, Ireland) to extract core images (4096 × 4096 pixels or 659.58 × 659.58 μm where 0.1613 μm corresponds to one pixel). We followed the instructions provided by Andrew Janowczyk to extract the core (spot) images from the whole slide TMA images (http://www.andrewjanowczyk.com/de-array-a-tissue-microarray-tma-using-qupath-and-python/; accessed 11/2022). Each core was categorized as malignant or benign.

Image preprocessing

To avoid unnecessary computational costs of large images, the core images or the marked prostate cancer area on whole slide images were tiled into small patches at 10× objective magnification level (512 × 512 pixels or ~330 × 330 μm) with a 50% overlap rate (i.e., 50% of the information content of two patches were similar). We considered the standard ISO RGB (red, green, and blue) color space with three color channels[11]. Instead of stain color normalization, we applied color space augmentation (i.e., manipulation of saturation, contrast, and brightness) to reduce the effect of color space on the model inference.

Supplementary table S1

Table S1: Cohort characteristics of the study population (CPCBN, Canada). The distribution of age at diagnosis, preoperative PSA level as well as pathological tumor stage and lymph node status did not significantly differ between development and external validation sets (p>0.05). However, we observed a significant association of the distribution of biochemical recurrence status (p=0.00310) and grade group (GG) at radical prostatectomy (RP) (P=0.01963) with the definition of the data set. We emphasize that our model was trained only on 319 cases with grade group I, III and IV, which represent two homogenous grade groups and one heterogenous grade group, as explained in the "Online Methods" section, and which was evaluated on the whole development set and external validation set. IQR: interquartile range; PSA: prostate-specific antigen; CI: confidence interval.

	Overall	Development set	External validation set
Patients, n (%)	1,489 (100)	600 (40.3)	889 (59.7)
Age at diagnosis, years, median (IQR)	62 (57–66)	61 (57–65)	62 (57–67)
Preoperative PSA level, ng/mL, mean (95% CI)	8.76 (8.32–9.19)	8.76 (8.19–9.33)	8.75 (8.13–9.38)
Grade groups at RP, n (%)			
GG1	479 (32.2)	209 (34.8)	270 (30.4)
GG2	595 (40.0)	250 (41.7)	345 (38.8)
GG3	224 (15.0)	77 (12.8)	147 (16.5)
GG4	112 (7.5)	33 (5.5)	79 (8.9)
GG5	79 (5.3)	31 (5.2)	48 (5.4)
Tumor stage, n (%)			
pT2	946 (63.5)	395 (65.8)	551 (62.0)
pT3a	382 (25.7)	152 (25.4)	230 (25.8)
pT3b	139 (9.3)	53 (8.8)	86 (9.7)
pT4	22 (1.5)	0 (0.0)	22 (2.5)
Lymph node status, n (%)			
pN0/X	1,443 (96.9)	586 (97.7)	857 (96.4)
pN1	46 (3.1)	14 (3.2)	32 (3.6)
Biochemical recurrence status, n (%)			
No	996 (66.9)	375 (62.5)	621 (69.9)
Yes	493 (33.1)	225 (37.5)	268 (30.1)
Prostate Cancer-specific death status, n (%)			
No	1,450 (97.4)	576 (96)	874 (98.3)
Yes	39(2.6)	24 (4)	15 (1.7)
Median core number per patient, n (range)	3 (1–8)	3 (1–8)	3 (1 –8)
Total cores (images), n (%)	4,479 (100)	1,699 (37.9)	2,780 (62.1)
Total patches, n (%)	40,311 (100)	15,291 (37.9)	25,020 (62.1)
Number of centers, n (%)	5 (100)	2 (40%)	3 (60%)

Supplementary table S2

Table S2: Cohort characteristics of the study population (PROCURE, Canada). IQR: interquartile range; CI: Confidence interval; CRPC: castration-resistant prostate cancer; RP: Radical prostatectomy.

	Overall
Patients, n (%)	897
Age at diagnosis, years, median (IQR)	62 (58–67)
Preoperative PSA level, ng/mL, mean (95% CI)	7.8 (7.28–8.32)
Grade groups at RP, n (%)	
GG1	143 (15.9)
GG2	455 (50.7)
GG3	204 (22.7)
GG4	27 (3.1)
GG5	68 (7.6)
Tumor stage, n (%)	
pT2	535 (59.6)
pT3a	252 (28.1)
pT3b	110 (12.3)
Lymph node status, n (%)	
pN0/X	858 (95.7)
pN1	39 (4.3)
Biochemical recurrence status, n (%)	
No	568 (63.3)
Yes	329 (36.7)
Time to biochemical recurrence in months, median (IQR)	21 (4–46)
Prostate Cancer-specific death status, n (%)	
No	876 (97.7)
Yes	21 (2.3)
Time to cancer-specific death in months, median (IQR)	95 (62–103)
Median core number per patient, n (range)	3 (1-7)
Total cores (images), n	2,673
Total patches, n (%)	24,057
CRPC development during follow-up, n (%)	47
Time to CRPC in months, median (IQR)	70 (53.5–94.0)
Number of centers, n (%)	4

Supplementary table S3

Table S3: Cohort characteristics of the third external validation set (PLCO, U.S.). Grade grouping according to Epstein et al. was not feasible to determine due to the impossibility to differentiate between Gleason scores 3+4 and 4+3 from the PLCO dataset. AJCC prostate pathologic stage system combines TNM classification system, Gleason score, preoperative PSA levels. No patients had any evidence for distant metastases nor received neoadjuvant hormone therapy. Patients were prospectively randomized for PSA screening and enrolled in 10 multinational U.S. centers. In all slides, PCa lesions were delineated according to the pathologist's instruction. Slides with PCa were representative of the index lesion for each patient. IQR: interquartile range; PSA: prostate-specific antigen; CI: confidence interval; RP: radical prostatectomy.

	Overall
Patients, n	861
Age at diagnosis, years, median (IQR)	61 (58–68)
Preoperative PSA level, ng/mL, mean (95% CI)	6.45 (6.14–6.76)
Pathologic tumor stage, n (%)	
pT2	670 (77.8)
pT3a/b	191 (22.2)
Lymph node status, n (%)	
pN0/x	861 (100.0)
Gleason score at RP	
Gleason score 6	443 (51.45)
Gleason score 7	324 (37.63)
Gleason score 8	53 (6.16)
Gleason score 9-10	41 (4.76)
AJCC Prostate Pathologic stage 7th version	
Stage I	20 (2.32)
Stage IIa	81 (9.41)
Stage IIb	435 (50.52)
Stage III	134 (15.56)
Stage IV	191 (22.18)
Prostate Cancer-specific death status, n (%)	
No	835 (96.98)
Yes	26 (3.02)
Follow-up duration for prostate cancer survivors in months, median (IQR)	167 (137–195)
Time to cancer-specific death in months, median (IQR)	116 (73–151)
Whole slide imaging	
One slide per patient, n (%)	322 (37.40)
Two slides per patient, n (%)	437 (50.75)
Three slides per patient, n (%)	102 (11.85)
Total whole slide images, n	1,502
Patches with prostate cancer per slide, median (IQR)	300 (78–735)
Total patches with prostate cancer	847,474

Supplementary table S4

Table S4 shows the results of comparative analyses between our model, classical and state-of-the-art models that are used in medical imaging. We provided the grade groups as reference. Our novel model delivered a higher generalized concordance probability, a larger effect size or z values than EfficientNet B1, VGG-16 and ResNet-50RS, although our model is computationally efficient (1/3 of EfficientNet B1, 1/76 of VGG-16 or 1/22 of ResNet-50RS), it used fewer features in the final convolutional layer, the last layer before the full connection of these features for prediction (1/20 of EfficientNet B1, 1/8 of VGG-16 or 1/32 of ResNet-50RS) and lesser parameter capacity (1/24 of EfficientNet B1, 1/54 of VGG-16 or 1/125 of ResNet-50RS) to compete with these existing models. We used the univariate weighted cox regression model to estimate generalized concordance probability, the effect size, the z value, the Akaike information criterion (AIC) and the Bayesian information criterion (BIC). The best measured metrics are highlighted in bold. The non-nested partial likelihood ratio test for cox regression models revealed that EfficientNet B1 does not fit better than our novel model (z=0.396 p = 0.346) by ΔAIC = ΔBIC = 5.594. The computation efficacy was measured using gFLOPS (giga floating point operations per second) on the input dimension (16×512×512×3 pixels). The lower gFLOPS, the more efficient is the model.

	Grade groups	our novel model	EfficientNet B1	VGG-16	ResNet50RS
Parameter capacity	-	**270,357**	6,576,520	14,715,201	33,698,337
Number of feature maps in the last convolutional layer	-	**64**	1,280	512	2,048
gFLOPS	-	**33.90**	98.55	2567.35	746.59
Concordance index	0.682±0.017	0.682±0.018	**0.688±0.018**	0.635±0.011	0.667±0.018
Generalized concordance probability	0.875 (0.771–0.936)	**0.927 (0.891–0.952)**	0.875 (0.837–0.905)	0.807 (0.748–0.854)	0.861 (0.818–0.896)
Effect size	7.000 (3.371–14.540)	**12.703 (8.178–19.729)**	6.995 (5.122–9.553)	4.171 (2.966–5.865)	6.205 (0.484–8.586)
z	5.218843	11.31463	**12.23275**	8.213045	11.01189
AIC	3365.577	3328.96	3323.366	3378.062	3351.338
BIC	3369.168	3332.551	3326.957	3381.653	3354.929

Supplementary table S5

Table S5 shows the results of comparative analyses for different objective magnification (10x, 20x and 40x), as well as the Cox deep model concept and the attention aggregation layer (as alternative for average pooling). These results demonstrate that considering objective magnification higher than 10x, attention aggregation layer instead of average pooling, as well as the cox deep model concept resulted in inferior performance. We used the univariate weighted cox regression model to estimate generalized concordance probability, the effect size, the z value, the Akaike information criterion (AIC) and the Bayesian information criterion (BIC).

	Our strategy	Alternative strategies			
	10x objective magnification	20x objective magnification	40x objective magnification	Attention aggregation layer	Cox deep model
Concordance index	**0.682±0.018**	0.660±0.018	0.557±0.018	0.610±0.011	0.588±0.018
Generalized concordance probability	**0.927 (0.891–0.952)**	0.861 (0.818–0.896)	n.c.	0.838 (0.756–0.896)	0.719 (0.627–0.797)
Effect size	**12.703 (8.178–19.729)**	6.205 (4.484–8.586)	n.c.	5.173 (3.095–8.646)	2.563(1.677–3.916)
z	**11.31463**	11.01189	3.201225	6.27067	4.35174
AIC	**3328.960**	3387.361	3460.59	3428.935	3446.709
BIC	**3332.551**	3390.952	3464.181	3432.526	3450.300

Supplementary table S6

*Table S6: Univariate and multivariate weighted Cox regression analyses for recurrence (BCR) in the first validation set (CPCBN, Canada). Age at diagnosis and positive surgical margins (PSM) were not significant nor independent predictors for BCR. Therefore, the multivariate analysis excluded both parameters. CI: confidence interval. z was only reported in the multivariate analysis. PSM: positive surgical margin (reference: negative). PSA: Prostate-specific antigen. IDC: Presence of intraductal carcinoma of the prostate (reference: absent). *Log- transformed.*

	Univariate analysis		Multivariate analysis			
Parameter	Hazard ratio (95% CI)	*p*	Hazard ratio (95% CI)	*z*	*p*	Generalized concordance probability (95% CI)
Age at diagnosis	1.01 (0.99–1.03)	0.1578	-	-	-	-
PSA level*	1.91 (1.50–2.44)	<0.0001	1.41 (1.12–1.76)	2.92	0.0345	0.505 (0.503–0.507)
IDC	1.83 (1.22–2.76)	0.0036	1.28 (0.79–2.09)	1.00	0.3150	0.566 (0.452–0.674)
PSM	0.75 (0.11–5.05)	0.7631	-	-	-	-
Tumor stage						
pT2	Reference		Reference			
pT3a	2.43 (1.80–3.29)	<0.0001	1.75 (1.27–2.43)	3.39	0.0071	0.643 (0.566–0.713)
pT3b	5.90 (4.21–8.26)	<0.0001	3.03 (2.07–4.43)	5.72	<0.0001	0.754 (0.676–0.818)
pT4	6.74 (3.41–13.30)	<0.0001	3.31 (1.29–8.52)	2.49	0.0128	0.750 (0.525–0.891)
Grade groups						
GG1	Reference		Reference			
GG2	1.91 (1.41–2.59)	<0.0001	1.37 (0.92–2.04)	1.55	0.1202	0.591 (0.493–0.682)
GG3	2.54 (4.36–8.71)	<0.0001	1.49 (0.95–2.34)	1.72	0.0848	0.614 (0.502–0.714)
GG4	4.96 (2.89–8.49)	<0.0001	1.52 (0.88–2.63)	1.51	0.1308	0.613 (0.479–0.731)
GG5	6.92 (3.16–15.15)	<0.0001	2.54 (1.47–4.42)	3.32	0.0090	0.731 (0.612–0.825)
**BCR score**	**12.70 (8.18–19.73)**	**<0.0001**	**6.43 (3.84–10.77)**	**7.07**	**<0.0001**	**0.867 (0.794–0.917)**

Supplementary table S7

*Table S7: multivariate weighted Cox regression analyses of the Grade Groups and the novel risk groups for biochemical recurrence (BCR) on the second validation set (PROCURE, Canada) in addition to preoperative prostate-specific antigen (PSA), tumor staging classification (pT and pN) and positive surgical margin. CI: confidence interval. The hazard ratios for BCR are calculated. CI: confidence interval. *Log- transformed.*

Parameter	Hazard ratio (95% CI)	*z*	*p*	Generalized concordance probability (95% CI)
PSA level*	1.55 (1.25–1.93)	4.03	<0.0001	0.609 (0.556–0.658)
Tumor stage				
pT2	Reference			
pT3a	2.00(1.48–2.71)	4.46	<0.0001	0.667 (0.596–0.731)
pT3b	3.23 (2.13–4.89)	5.53	<0.0001	0.764 (0.681–0.830)
Nodal stage				
pN1 vs. pN0/x	1.48 (0.83–2.64)	1.34	0.1818	0.597 (0.454–0.725)
Surgical margin status				
Positive vs. Negative	2.29 (1.69–3.09)	5.38	<0.0001	0.696 (0.629–0.756)
Grade Groups				
GG1	Reference			
GG2	2.45 (1.18–5.13)	2.82	0.0169	0.711 (0.540–0.837)
GG3	3.67 (1.74–7.76)	3.40	0.0006	0.786 (0.634–0.886)
GG4	5.15 (2.08–12.77)	3.54	0.0004	0.837 (0.675–0.927)
GG5	8.12 (3.54–18.63)	4.94	<0.0001	0.890 (0.830–0.959)
BCR score-based risk groups				
Low	Reference			
Low-intermediate	3.44 (1.99–5.94)	4.44	<0.0001	0.775 (0.666–0.856)
High-intermediate	6.84 (3.80–12.30)	6.42	<0.0001	0.872 (0.792–0.925)
High	12.73 (6.91–23.46)	8.16	<0.0001	0.927 (0.874–0.959)

Supplementary table S8

Table S8: Biochemical recurrence (BCR)-free survival probabilities for all risk groups based on BCR scores at different time points in the external validation sets (CPCBN and PROCURE). CI: confidence interval.

BCR score-based risk groups	BCR-free survival rates		
	3rd year (95% CI)	5th year (95% CI)	10th year (95% CI)
**1st external validation set (CPCBN)** (log-rank $p < 0.0001$)			
Low	92.1 (87.5–96.9)	90.4 (85.3–95.7)	88.2 (82.5–94.3)
Low-intermediate	86.8 (83.8–90.0)	83.1 (79.8–86.7)	75.6 (71.3–80.2)
High-intermediate	78.1 (72.2–84.4)	69.7 (63.2–76.9)	54.0 (45.6–63.8)
High	54.0 (45.5–64.0)	36.7 (28.8–46.9)	23.7 (16.1–35.1)
**2nd external validation set (PROCURE)** (log-rank $p < 0.0001$)			
Low	94.0 (90.6–97.5)	91.6 (87.6–95.8)	85.7 (79.2–92.8)
Low-intermediate	79.2 (75.8–82.7)	71.7 (67.9–75.7)	64.4 (60.1–69.0)
High-intermediate	44.9 (37.3–54.2)	37.3 (29.8–46.6)	28.5 (21.0–38.7)
High	19.4 (9.4–39.7)	9.7 (3.3–28.4)	-

Supplementary table S9

Table S9: recurrence-free survival rates for all grade groups based at different time points in the first external validation set (CPCBN, Canada). CI: confidence interval.

Grade Groups	Recurrence cases	Recurrence-free survival rates (log rank p<0.0001)		
	n (%)	3rd year (95% CI)	5th year (95% CI)	10th year (95% CI)
GG1	40 (14.8)	93.3 (90.3–96.3)	88.9 (85.1–92.8)	83.0 (77.9–88.4)
GG2	90 (26.1)	85.9 (82.2–89.6)	79.9 (75.7–84.3)	68.6 (62.8–74.9)
GG3	61 (41.5)	73.9 (67.1–81.4)	68.8 (61.7–76.8)	53.3 (44.6–63.6)
GG4	45 (56.9)	58.0 (48.1–70.0)	45.8 (35.9–58.5)	42.2 (32.2–55.3)
GG5	32 (66.7)	47.9 (35.7–64.4)	37.5 (26.0–54.0)	17.3 (10.1–29.8)

Supplementary table S10

Table S10: recurrence-free survival rates for all grade groups based at different time points in the second external validation set (PROCURE, Canada). CI: confidence interval.

Grade Groups	Recurrence cases	Recurrence-free survival rates (log rank p<0.0001)		
	n (%)	3rd year (95% CI)	5th year (95% CI)	10th year (95% CI)
GG1	15 (10.5)	95.7 (92.5–99.1)	94.2 (90.3–98.2)	88.5 (82.8–94.4)
GG2	130 (28.6)	81.9 (78.4–85.6)	75.9 (71.9–80.1)	62.9 (56.6–70.0)
GG3	111 (54.4)	64.7 (58.4–71.6)	54.8 (48.2–62.3)	34.8 (27.2–44.6)
GG4	17 (63.0)	48.1 (32.6–71.2)	37.0 (22.6–60.6)	37.0 (22.6–60.6)
GG5	56 (82.4)	24.5 (16.1–37.3)	17.3 (10.1–29.8)	-

Supplementary table S11

Table S11: Univariate and multivariate Cox regression analyses for cancer-specific mortality on the first external validation set (CPCBN, Canada). CI: confidence interval. Loco-regional lymph node status was not significant in univariate and multivariate analyses and therefore not presented here. Please note that c-index is not suitable for Cox model comparison.

	Univariate analysis				Multivariate analysis			
Parameter	Hazard ratio (95% CI)	*p*	c-index	AIC (BIC)	Hazard ratio (95% CI)	*z*	*p*	c-index
Tumor stage	2.58 (1.58–4.21)	0.0001	0.788	171.0623 (171.7703)	1.89 (1.07–3.34)	2.20	0.0275	0.850
Grade Groups	1.92 (1.30–2.84)	0.0010	0.670	173.2988 (174.0069)	1.33 (0.85–2.09)	1.24	0.2164	
Risk score	2.58 (1.47–4.55)	0.0010	0.767	172.3737 (173.0817)	1.83 (1.002–3.35)	1.97	0.0491	

Supplementary table S12

Table S12: univariate and multivariate weighted Cox regression analyses for tumor and nodal stages and the risk scores (continuous parameter) on the second external validation set (PROCURE, Canada). The hazard ratios for cancer-specific mortality are calculated. Due to the limited cancer-specific death events, we binarized the tumor stage (pT2 vs. pT3). The c-index for multivariate analysis is 0.921. CI: confidence interval. Please note that c-index is not suitable for Cox model comparison.

	Univariate analysis				Multivariate analysis+			
Parameter	Hazard ratio (95% CI)	*p*	c-index	AIC (BIC)	Hazard ratio (95% CI)	*z*	*p*	Generalized concordance probability
Tumor stage								
pT3 vs. pT2	1.97 (0.32 – 12.17)	0.4632	0.744	207.54 (208.58)	0.30 (0.02–4.10)	-0.91	0.3654	0.230 (0.021–0.804)
Nodal stage								
pN1 vs. pN0/x	21.43 (6.80 – 67.56)	<0.0001	0.622	202.32 (203.36)	8.25 (1.36–49.91)	2.30	0.0216	0.892 (0.577–0.980)
Grade Groups	76.06 (9.18 – 630.43)	<0.0001	0.866	183.06 (184.11)	12.84 (0.87 – 189.84)	1.86	0.0630	0.928 (0.465–0.995)
Risk score	109.06 (26.42 – 450.16)	<0.0001	0.867	185.06 (186.11)	64.69 (2.20 – 898.43)	2.42	0.0156	0.985 (0.688–1.000)

Supplementary table S13

Table S13: univariate and multivariate Cox regression analyses for cancer-specific mortality on the third external validation set (PLCO, U.S.). Note: These Gleason scores were determined prior the introduction of the 2014/2016 ISUP Gleason grading.

	Univariate analysis				Multivariate analysis			
Parameter	Hazard ratio (95% CI)	*p*	c-index	AIC (BIC)	Hazard ratio (95% CI)	*Z*	*p*	Generalized concordance probability
Gleason score	54.83 (18.35–163.83)	<0.0001	0.806	304.5 (305.8)	34.77 (12.40–97.52)	6.744	<0.0001	0.972 (0.925–0.990)
Risk score	33.36 (8.70–127.94)	<0.0001	0.738	315.8 (317.1)	16.17 (4.60–56.81)	4.341	<0.0001	0.942 (0.822–0.983)

Supplementary table S14

Table S14 reveals the results of Fine-Gray competing risk regression model for cancer-specific mortality (sub-distribution hazard ratios) on the first external validation set (CPCBN, Canada). HR reflects the ordering of the risk groups. The extreme high HRs for the risk groups are due to absent of cancer-specific death cases in the low-risk group. The number of death cases from causes other than Prostate Cancer was 72.*

Characteristic	HR	95% CI	p-value
Grade Groups			
GG1	—	—	
GG2	0.33	(0.06 – 2.01)	0.2
GG3	0.56	(0.09 – 3.42)	0.5
GG4	0.41	(0.06 – 2.82)	0.4
GG5	2.01	(0.31 – 13.0)	0.5
Tumor stage			
T2	—	—	
T3a	4.94	(0.92 – 26.5)	0.063
T3b	10.4	(1.56 – 68.9)	0.015
T4	5.28	(0.27 – 104)	0.3
The risk groups*			
Low	—	—	
Low-intermediate	11,850	(4,342 – 32,343)	<0.001
High-intermediate	10,871	(2,875 – 41,103)	<0.001
High	32,320	(9,682 – 107,892)	<0.001
HR = Hazard Ratio, CI = Confidence Interval			

Supplementary table S15

Table S15 reveals the results of Fine-Gray competing risk regression model for cancer-specific mortality (sub-distribution hazard ratios) on the second external validation set (PROCURE, Canada). HR reflects the ordering of the risk groups. The extreme high HRs for the risk groups and Grade groups are due to absent of cancer-specific death cases in the low-risk group and GG1. The number of death cases from causes other than Prostate Cancer was 69.*

**Characteristic**	**HR**	**95% CI**	**p-value**
Grade Groups			
GG1	—	—	
GG2	6,111	(2,630 – 14,200)	<0.001
GG3	3,917	(771 – 19,903)	<0.001
GG4	1,470	(29.4 – 73,470)	<0.001
GG5	11,532	(1,413 – 94,142)	<0.001
Tumor stage			
T2	—	—	
T3a	0.82	(0.18 – 3.71)	0.8
T3b	2.60	(0.71 – 9.55)	0.2
Lymph node stage			
pN0/x	—	—	
pN1	0.40	(0.13 – 1.26)	0.12
The risk groups*			
Low	—	—	
Low-intermediate	6,797	(3,002 – 15,390)	<0.001
High-intermediate	17,425	(3,549 – 85,551)	<0.001
High	106,639	(18,456 – 616,165)	<0.001
HR = Hazard Ratio, CI = Confidence Interval			

Supplementary table S16

Table S16 reveals the results of Fine-Gray competing risk regression model for cancer-specific mortality (sub-distribution hazard ratios) on the third external validation set (PLCO, US). The extreme high HRs for the risk groups and pathologic Prostate Stage are due to absent of cancer-specific death cases in the low-risk group and Stage I. The number of death cases from causes other than Prostate Cancer was 179.

**Characteristic**	**HR**	**95% CI**	**p-value**
Gleason score			
6	—	—	
7	5.92	(1.27 – 27.5)	0.023
8 – 9	22.1	(3.63 – 134)	<0.001
10	16.5	(2.48 – 110)	0.004
Pathologic Prostate Stage			
Stage I	—	—	
Stage IIA	0.80	(0.15 – 4.12)	0.8
Stage IIB	16,481	(4,634 – 58,614)	<0.001
Stage III	17,348	(5,580 – 53,935)	<0.001
Stage IV	24,404	(8,783 – 67,808)	<0.001
The novel risk groups*			
Low	—	—	
Low-intermediate	12,156	(5,759 – 25,656)	<0.001
High-intermediate	29,687	(14,108 – 62,469)	<0.001
High	35,406	(9,912 – 126,466)	<0.001
HR = Hazard Ratio, CI = Confidence Interval			

Supplementary table S17

Table S17: Cancer-specific survival rates for all risk groups at different time points in three external validation sets. χ^2 test shows a significant difference for the cancer-specific death status between the risk groups. The low-risk group has no cancer-specific death cases, resulting in the most favorable cancer-specific survival rates of 100%. SE, Standard Error. In the PROCURE cohort, estimating 15-year cancer-specific survival rates was not feasible due to the constraint of the follow-up duration.

Risk groups	Cancer-specific death cases	Cancer-specific survival rates	
	n (%)	10th year (SE)	15th year (SE)
**1st external validation set (CPCBN)** (log-rank p = 0.0014; χ^2(3, N=889) = 17.121, p = 0.0007)			
Low	0 (0)	100.0 (0)	100.0 (0)
Low-intermediate	5 (1.1)	99.3 (0.5)	96.8 (1.5)
High-intermediate	3 (1.7)	98.2 (1.3)	96.1 (2.9)
High	7 (6.2)	92.7 (2.3)	89.0 (4.6)
**2nd external validation set (PROCURE)** (log-rank p < 0.0001; χ^2(3, N=897) = 67.063, p <0.0001)			
Low	0 (0)	100.0 (0)	n.c.
Low-intermediate	7 (1.3)	96.6 (1.7)	n.c.
High-intermediate	7 (5.1)	72.8 (9.3)	n.c.
High	7 (22.6)	60.3 (14.1)	n.c.
**3rd external validation set (PLCO)** (log-rank p < 0.0001; χ^2(3, N=861) = 19.023, p = 0.0003)			
Low	0 (0)	100.0 (0)	100.0 (0)
Low-intermediate	11 (2.0)	98.9 (0.5)	97.8 (0.7)
High-intermediate	12 (6.3)	95.5 (1.5)	93.0 (2.1)
High	3 (12.5)	95.7 (4.3)	81.2 (10.2)

Supplementary table S18

Table S18: Multivariate Cox regression analysis including risk scores and the slide number on the third external validation set (PLCO, U.S.). Hazard ratio for cancer-specific mortality is estimated. The results indicate no significant interaction between the risk score and the slide number; the Pearson correlation coefficient between the risk score and the slide number was 0.005 (95% CI: -0.062–0.072; p=0.8792).

Parameter	Hazard ratio (95% CI)	*z*	*p*
Risk score	52.93 (2.47–1135.56)	2.537	0.0112
Slide number	1.91 (0.91–4.00)	1.710	0.0872
**Slide number · risk score**	**0.89 (0.19–4.12)**	**-0.146**	**0.8839**

Supplementary table S19

Table S19: cancer-specific survival rates for all grade groups based at different time points in the first external validation set (CPCBN, Canada). SE: Standard Error.

Grade Groups	Cancer-specific death cases	Cancer-specific survival rates (log rank p<0.0001)	
	n (%)	10th year (SE)	15th year (SE)
GG1	3 (1.1)	99.2 (0.6)	98.0 (1.3)
GG2	2 (0.6)	99.3 (0.6)	98.0 (1.5)
GG3	3 (2.0)	98.0 (1.4)	96.1 (2.3)
GG4	2 (2.5)	100 (0)	92.2 (5.3)
GG5	5 (10.4)	83.0 (7.0)	83.0 (7.0)

Supplementary table S20

Table S20: cancer-specific survival rates for all grade groups based at different time points in the second external validation set (PROCURE, Canada). SE: Standard Error.

Grade Groups	Cancer-specific death cases	Cancer-specific survival rates (log rank p<0.001)
	n (%)	10th year (SE)
GG1	0 (100)	100 (0)
GG2	6 (1.3)	95.5 (2.1)
GG3	4 (2.0)	96.4 (2.9)
GG4	1 (3.7)	93.8 (6.1)
GG5	10 (14.7)	45.2 (14.2)

Supplementary table S21

Table S21: Cancer-specific survival rates for Gleason scores at different time points in the third external validation set (PLCO, U.S.). These Gleason scores were determined according to prior grading guidelines because the PLCO patients were staged and graded between 1993 and 2008. SE: Standard Error.

Gleason score	Cancer-specific death cases	Cancer-specific survival rates (log rank p<0.0001)	
	n (%)	10th year (SE)	15th year (SE)
GS 6	2 (0.45)	99.8 (0.24)	99.4 (0.40)
GS 7	12 (3.70)	98.3 (0.74)	95.6 (1.41)
GS 8	7 (13.21)	91.8 (3.96)	82.4 (6.43)
GS 9-10	5 (12.20)	87.9 (5.70)	80.6 (8.74)

Supplementary figure S1

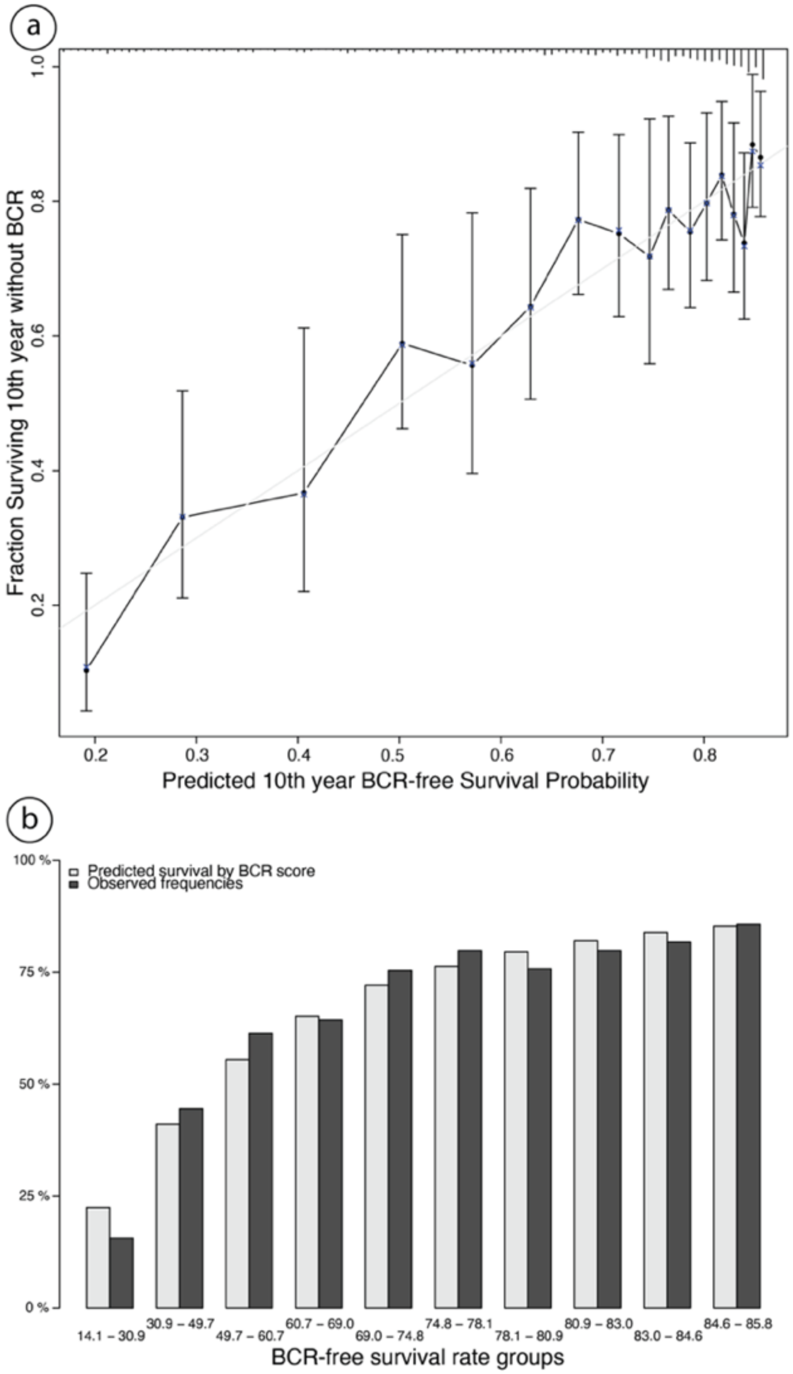

Figure 1: a. The calibration plot for our biochemical recurrence (BCR)-free prediction model at the 10th year with the rug plot (upper line) for distribution visualization. b. The histogram for observed and predicted survival by BCR scores within groups defined by deciles for observed BCR-free survival rates in the first external Validation set.

Supplementary figure S2

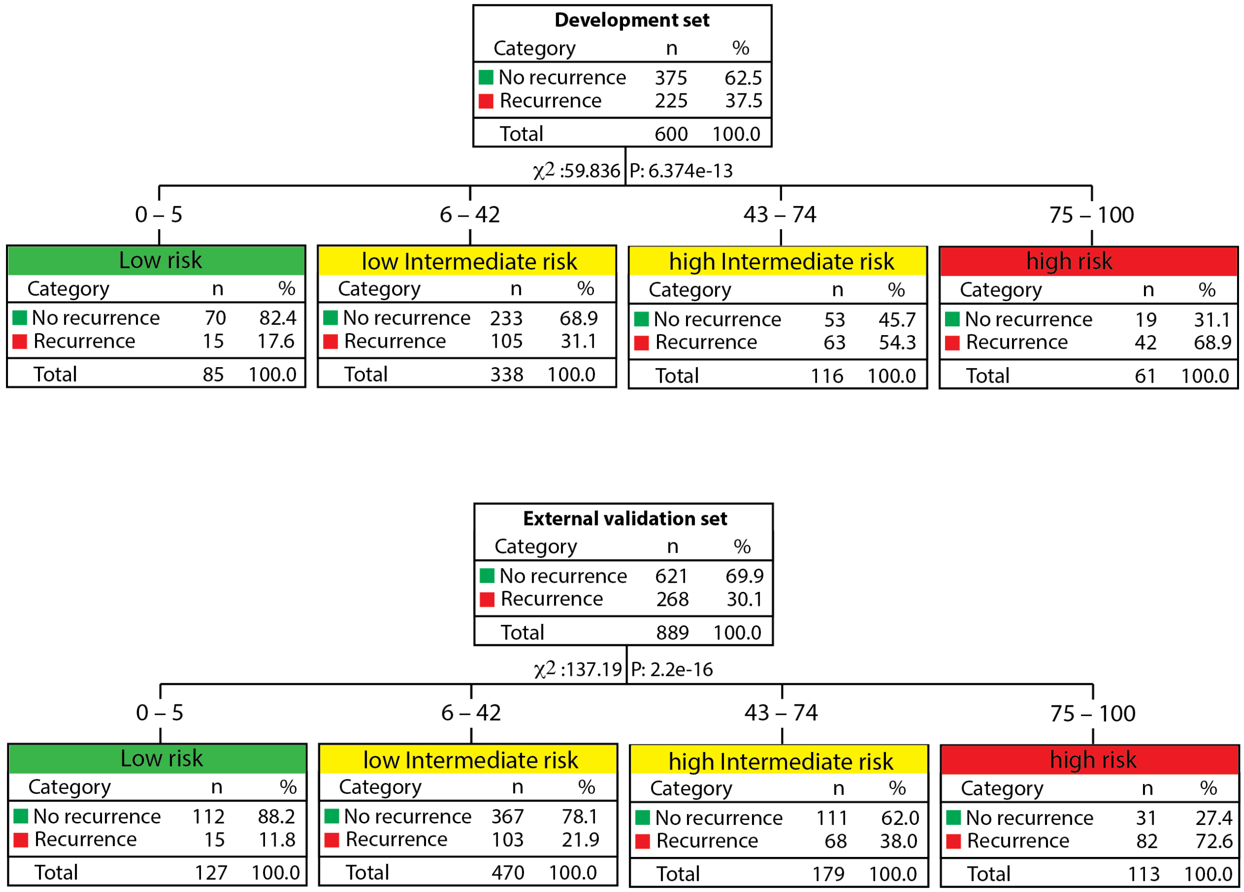

Figure 2: Risk strata according to the BCR score and corresponding BCR-free rates. Chi-square Automatic Interaction Detection (CHAID) was applied to the Development set to determine the cutoffs for these risk groups. The resulting cutoffs for the BCR scores were fixed for the external Validation set. X^2 represents Chi-square statistics.

Supplementary figure S3

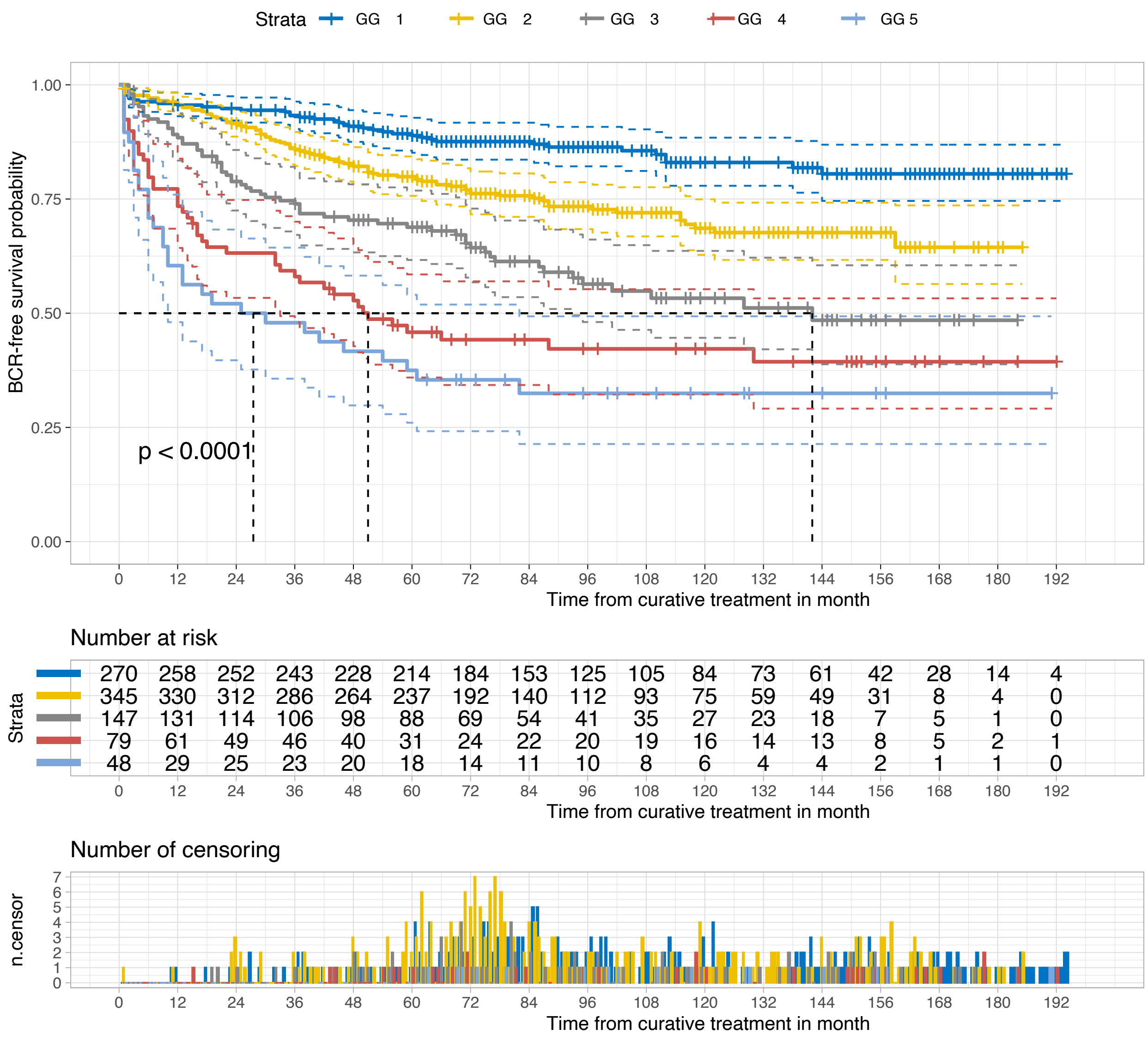

Figure S3: Kaplan-Meier curves of biochemical recurrence (BCR)-free survival according to grade groups (GG) in the first external validation set (CPCBN, Canada). The dotted lines indicate the median survival. The p-value was measured using the log-rank test. The number of patients at risk and of censored observations are provided for the follow-up period.

Supplementary figure S4

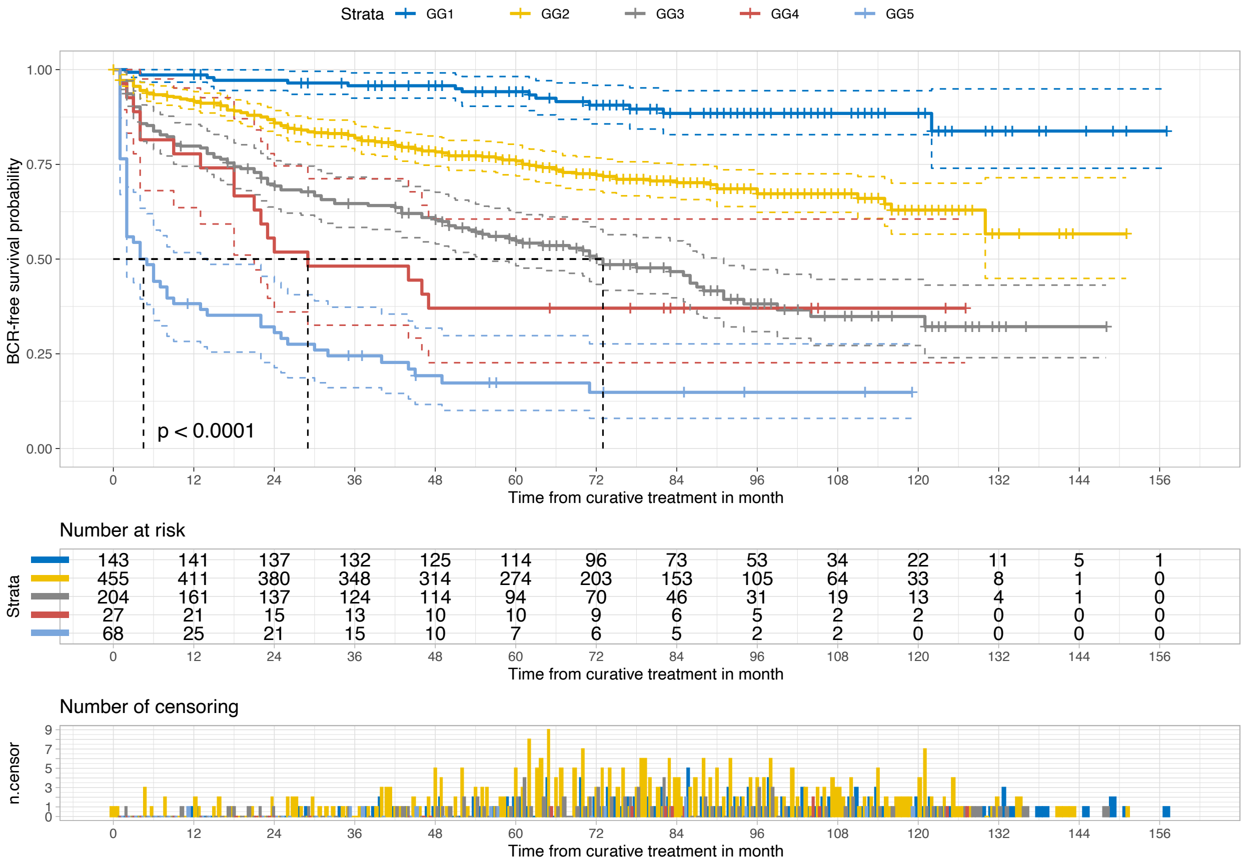

Figure S4: Kaplan-Meier curves of biochemical recurrence (BCR)-free survival stratified by grade groups (GG) in the second external validation set (PROCURE, Canada). The dotted lines indicate the median survival. The p-value was measured using the log-rank test. The number of patients at risk and of censored observations are provided for the follow-up period.

Supplementary figure S5

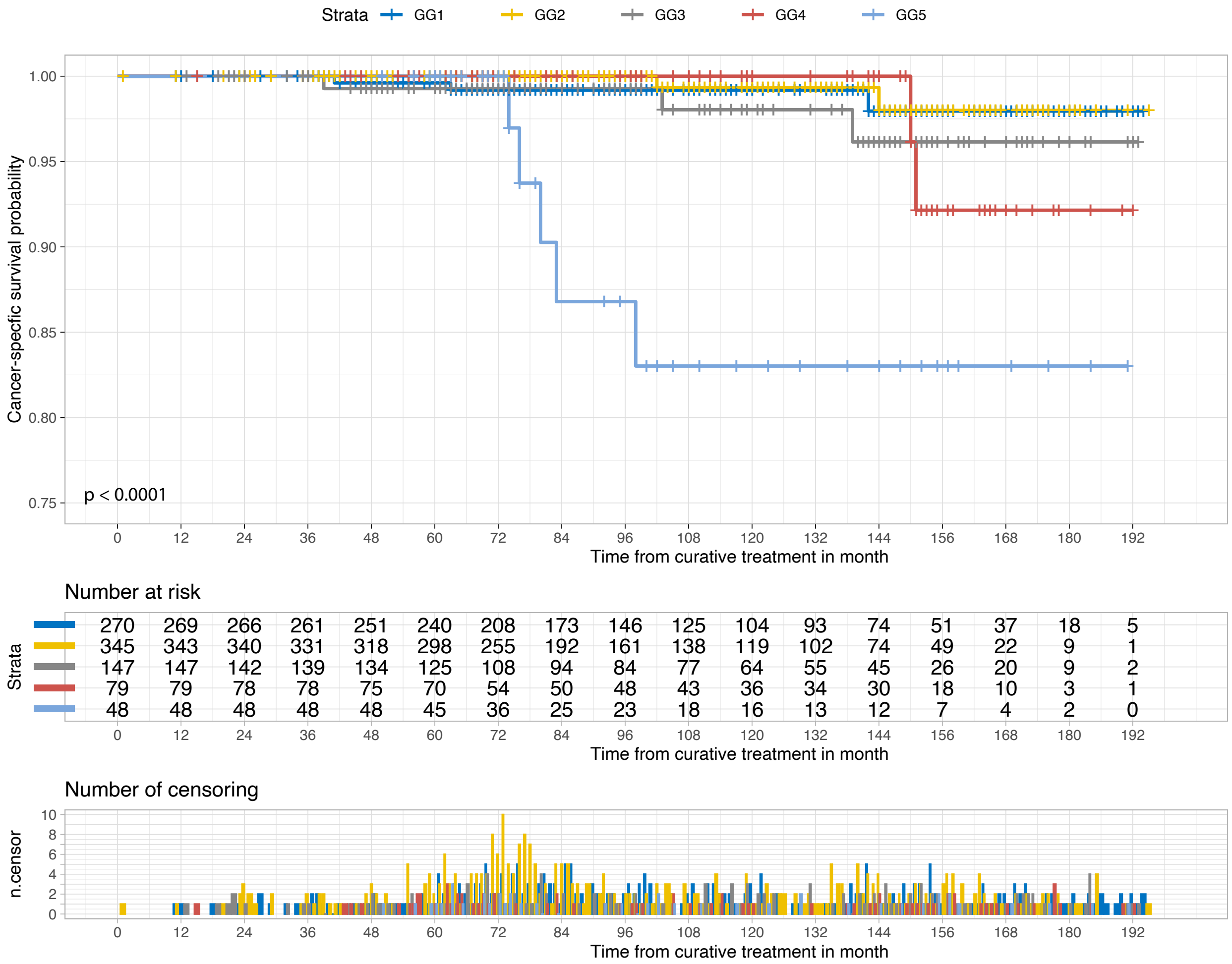

Figure S5: Kaplan-Meier curves of cancer-specific survival stratified by grade groups (GG) in the first external validation set (CPCBN, Canada)The p-value was measured using the log-rank test. The number of patients at risk and of censored observations are provided for the follow-up period.

Supplementary figure S6

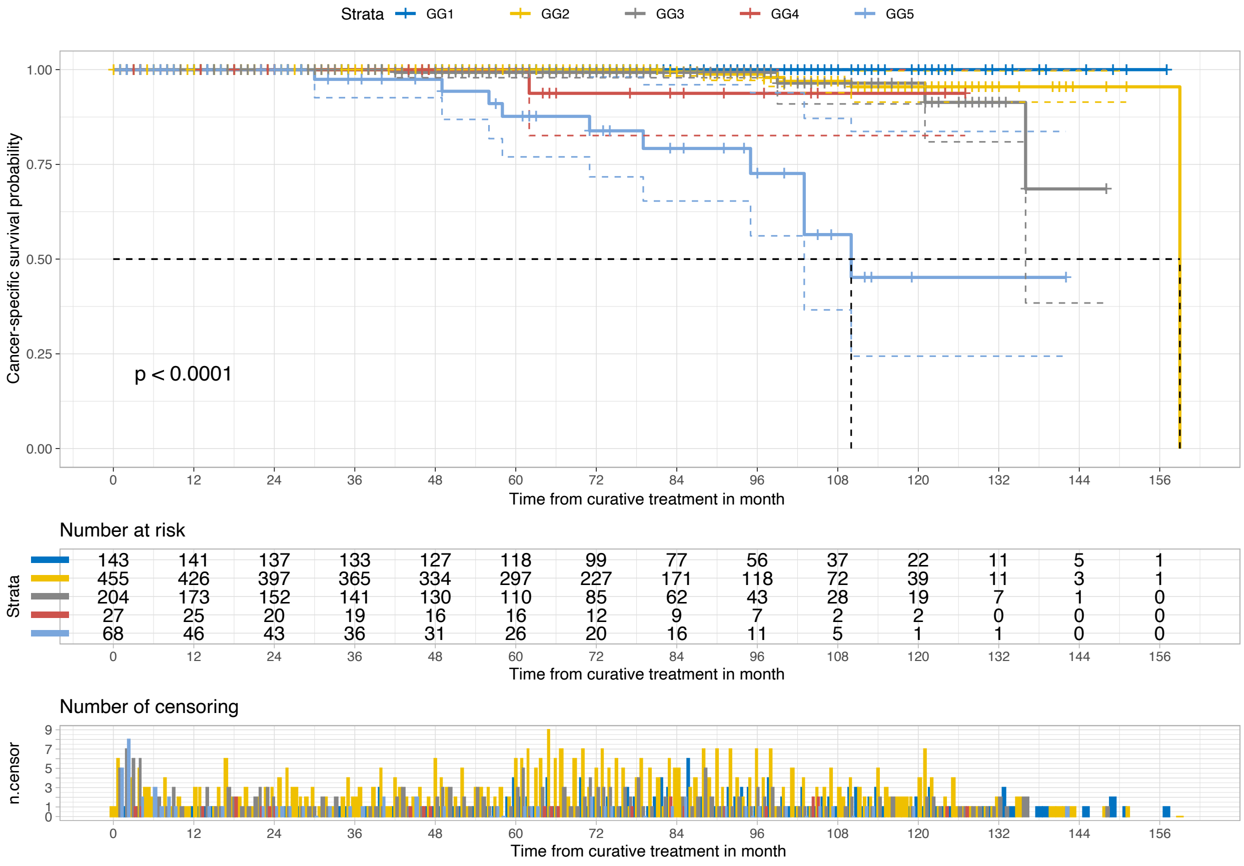

Figure S6: Kaplan-Meier curves of cancer-specific survival stratified by grade groups (GG) in the second external validation set (PROCURE, Canada). The p-value was measured using the log-rank test. The number of patients at risk and of censored observations are provided for the follow-up period.

Supplementary figure S7

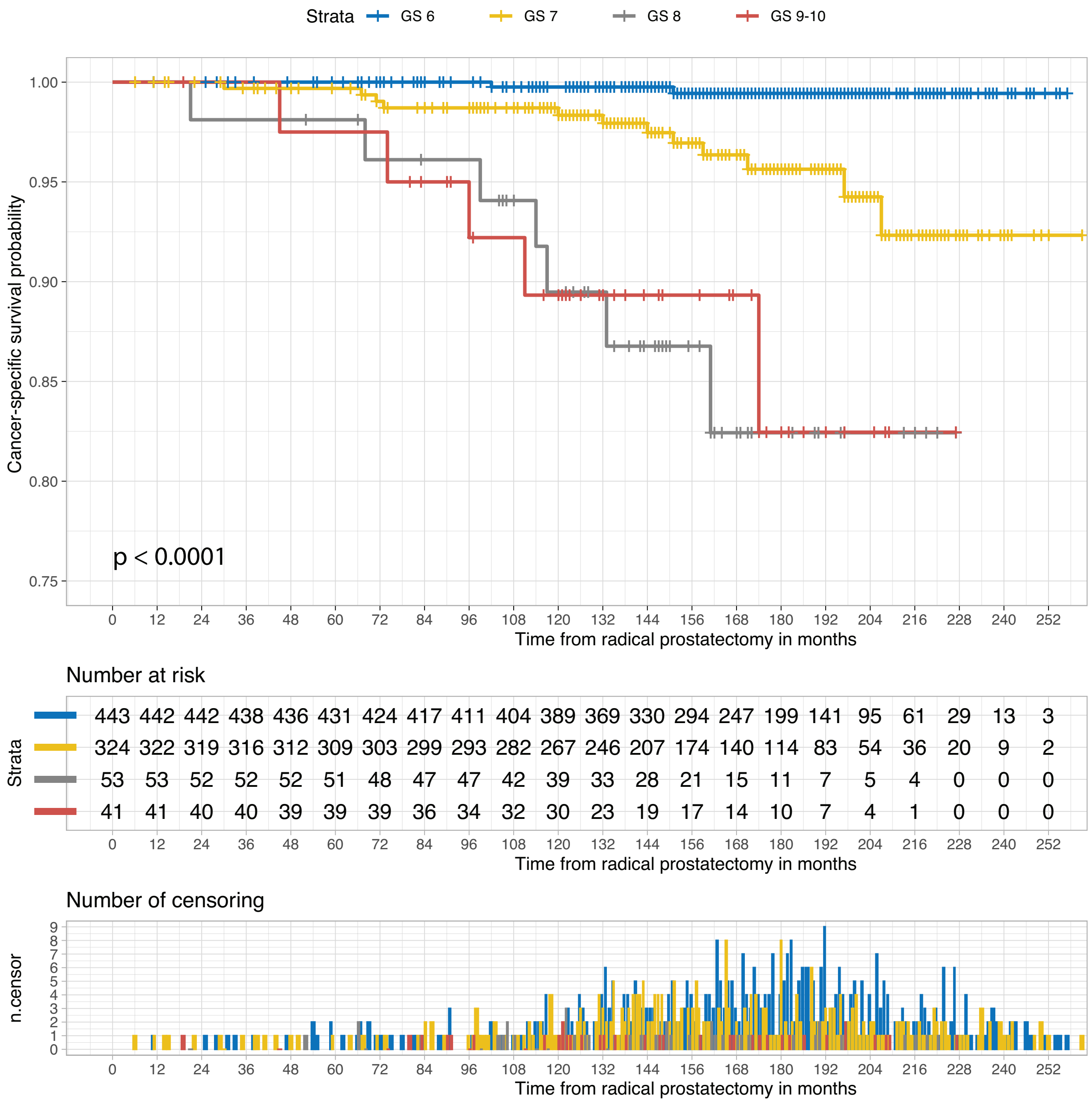

Figure S7: Kaplan-Meier curves of cancer-specific survival stratified by Gleason scores (GS) in the third external validation set (PLCO, U.S.). The p-value was measured using the log-rank test. The number of patients at risk and of censored observations are provided for the follow-up period.

Supplementary figure S8

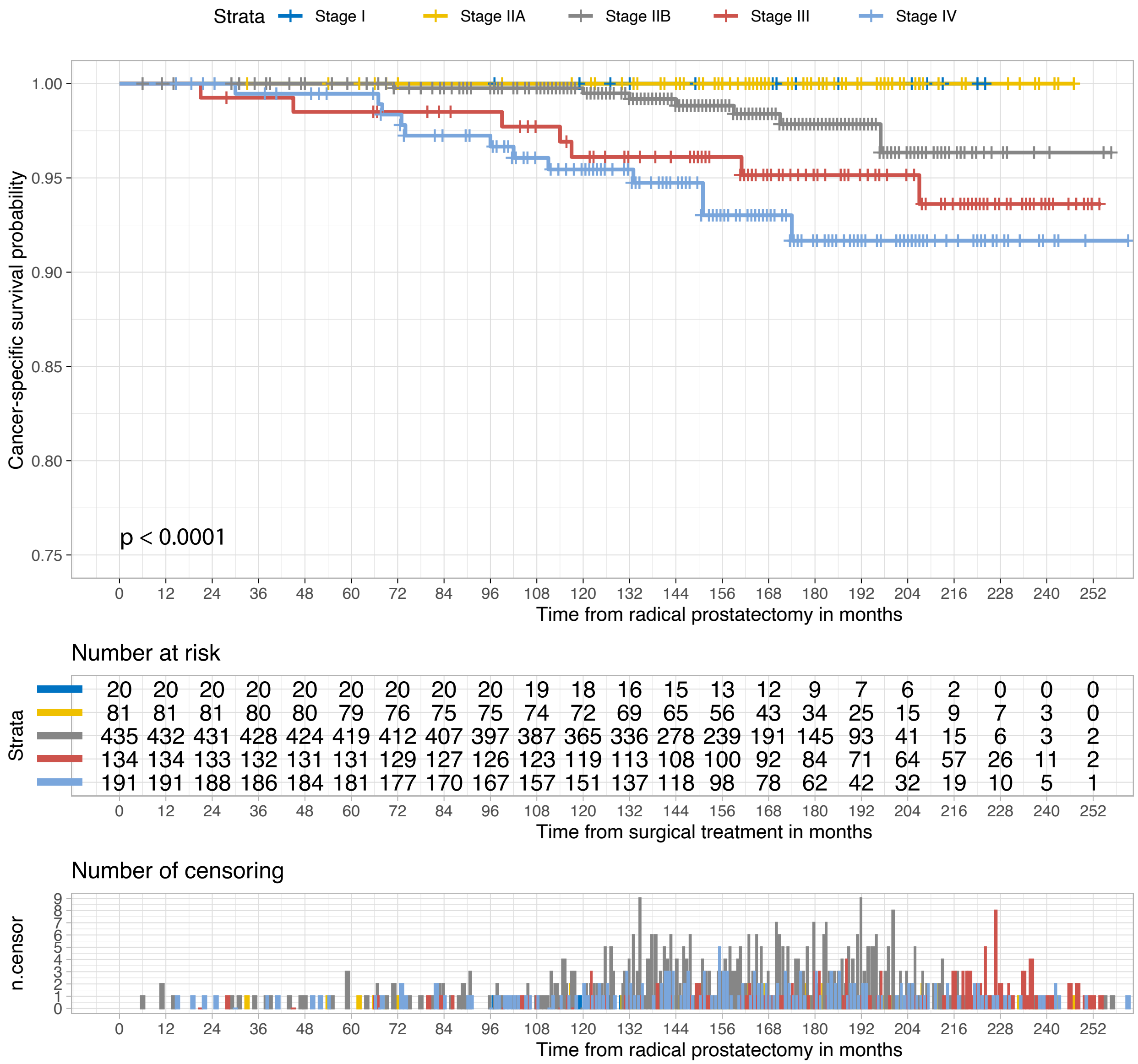

Figure S8: Kaplan-Meier curves of cancer-specific survival stratified by prostate cancer pathologic stage in the third external validation set (PLCO, U.S.). The p-value was measured using the log-rank test. The number of patients at risk and of censored observations are provided for the follow-up period.

Supplementary figure S9

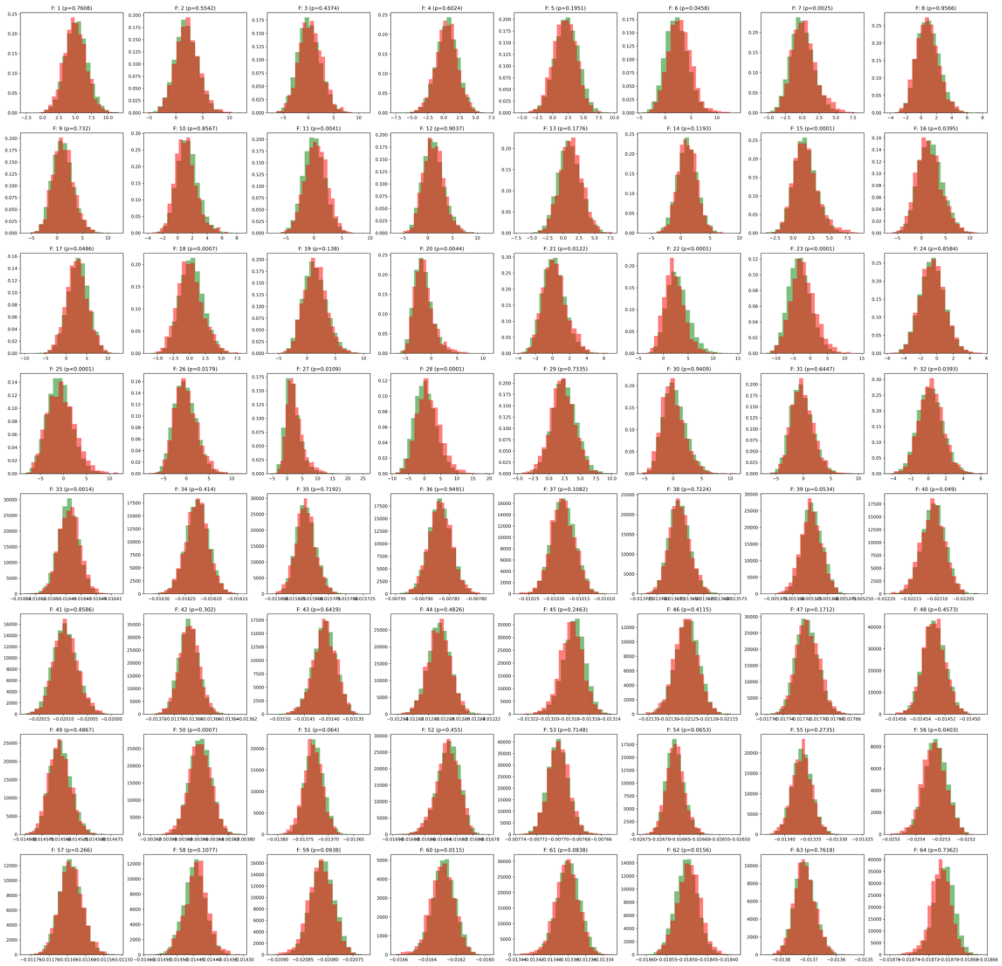

Figure S9: Histograms for 64 feature representations stratified by recurrence status (red: BCR; green: no BCR). The overlapped distribution is highlighted in brown. The Levene test was used to estimate p-values. The variance difference is significant when p≤0.00010.Five feature representations (F15, F22, F23, F25) have significant difference in feature distribution between BCR and nonBCR.

Supplementary figure S10

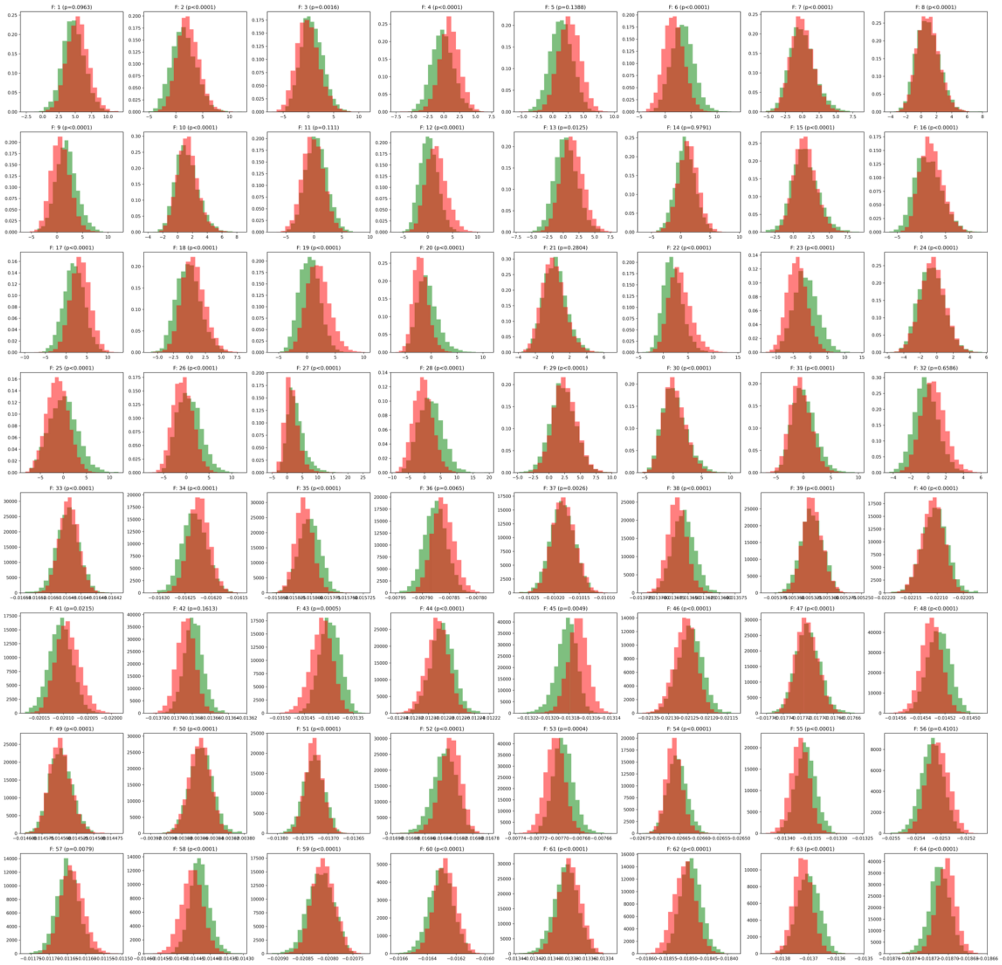

Figure S10: Histograms for 64 feature representations stratified by low-risk group status (red: low-risk group; green: other groups). The overlapped distribution is highlighted in brown. The Levene test was used to estimate p-values. The variance difference is significant when p≤0.0001.

Supplementary figure S11

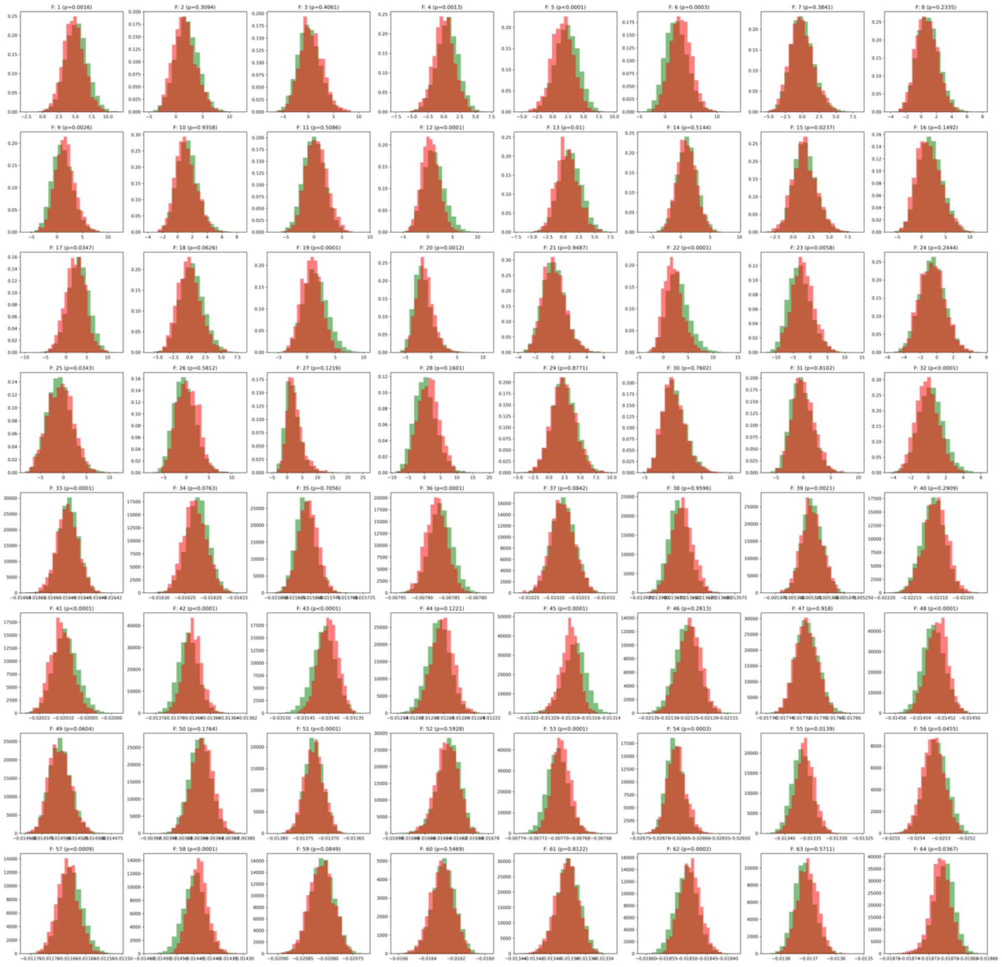

Figure S11: Histograms for 64 feature representations stratified by low intermediate-risk group status (red: low intermediate-risk group; green: other groups). The overlapped distribution is highlighted in brown. The Levene test was used to estimate p-values. The variance difference is significant when p≤0.0001.

Supplementary figure 12

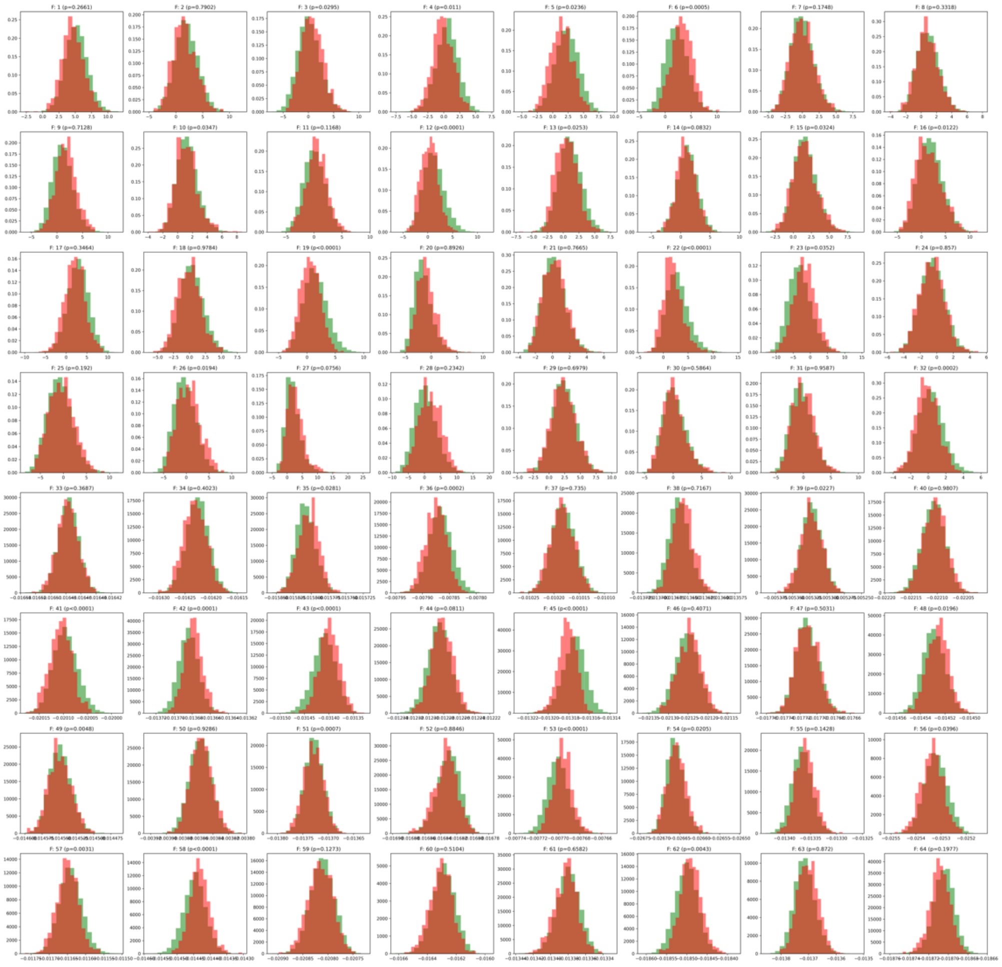

Figure S12: Histograms for 64 feature representations stratified by high intermediate-risk group status (red: high intermediate-risk group; green: other groups). The overlapped distribution is highlighted in brown. The Levene test was used to estimate p-values. The variance difference is significant when p≤0.0001.

Supplementary figure 13

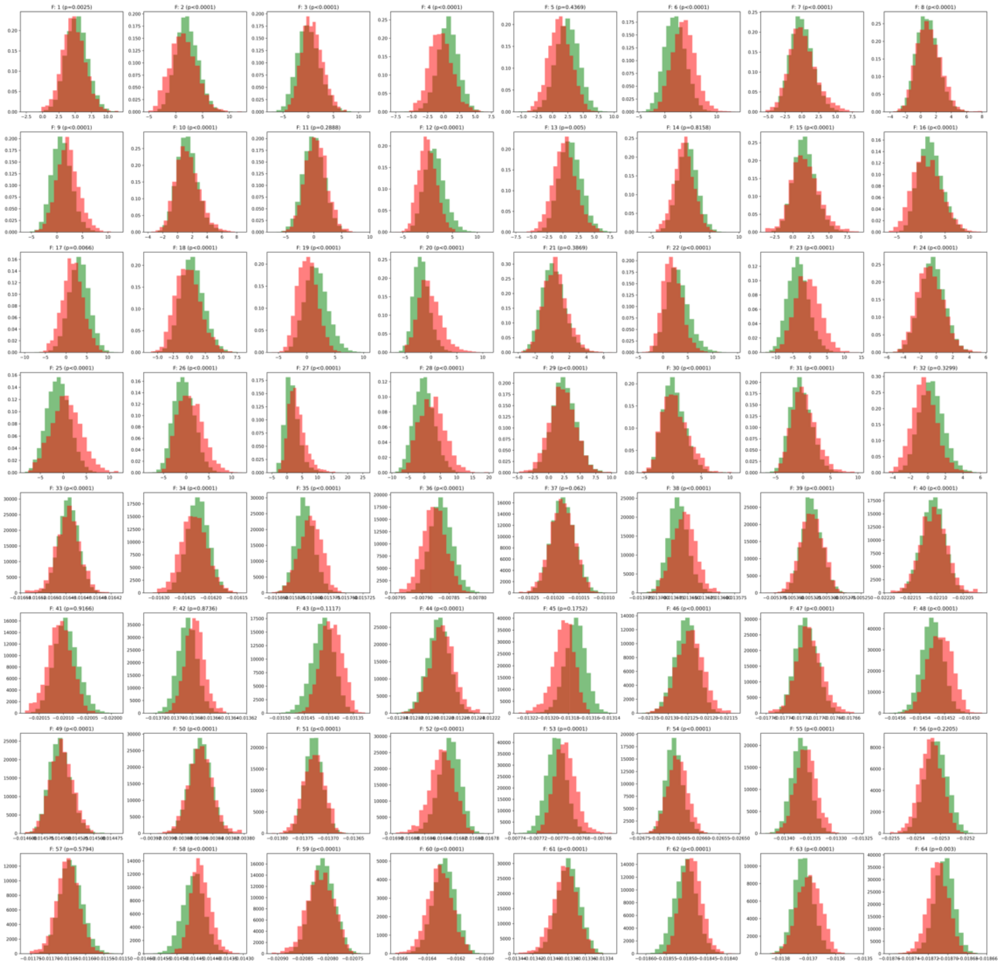

Figure S13: Histograms for 64 feature representations stratified by high-risk group status (red: high-risk group; green: other groups). The overlapped distribution is highlighted in brown. The Levene test was used to estimate p-values. The variance difference is significant when p≤0.0001.

References

1. Ouellet V, Aprikian A, Bergeron A, et al. The Terry Fox Research Institute Canadian Prostate Cancer Biomarker Network: an analysis of a pan-Canadian multi-center cohort for biomarker validation. *BMC Urol* 2018; **18**(1): 78.
2. Leyh-Bannurah SR, Trudel D, Latour M, et al. A Multi-Institutional Validation of Gleason Score Derived from Tissue Microarray Cores. *Pathol Oncol Res* 2019; **25**(3): 979-86.
3. Greene FL, Balch CM, Fleming ID, et al. AJCC cancer staging handbook: TNM classification of malignant tumors: Springer Science & Business Media; 2002.
4. Egevad L, Delahunt B, Srigley JR, Samaratunga H. International Society of Urological Pathology (ISUP) grading of prostate cancer - An ISUP consensus on contemporary grading. *APMIS* 2016; **124**(6): 433-5.
5. Wissing M, Brimo F, Chevalier S, et al. Optimization of the 2014 Gleason grade grouping in a Canadian cohort of patients with localized prostate cancer. *BJU Int* 2019; **123**(4): 624-31.
6. Brimo F, Aprikian A, Latour M, et al. Strategies for biochemical and pathologic quality assurance in a large multi-institutional biorepository; The experience of the PROCURE Quebec Prostate Cancer Biobank. *Biopreserv Biobank* 2013; **11**(5): 285-90.
7. Bankhead P, Loughrey MB, Fernández JA, et al. QuPath: Open source software for digital pathology image analysis. *Scientific reports* 2017; **7**(1): 1-7.
8. Team PP, Gohagan JK, Prorok PC, Hayes RB, Kramer B-S. The prostate, lung, colorectal and ovarian (PLCO) cancer screening trial of the National Cancer Institute: history, organization, and status. *Controlled clinical trials* 2000; **21**(6): 251S-72S.
9. Andriole GL, Crawford ED, Grubb III RL, et al. Mortality results from a randomized prostate-cancer screening trial. *New England journal of medicine* 2009; **360**(13): 1310-9.
10. Pinsky PF, Prorok PC, Yu K, et al. Extended mortality results for prostate cancer screening in the PLCO trial with median follow-up of 15 years. *Cancer* 2017; **123**(4): 592-9.
11. Saini HS, Sayal R, Buyya R, Aliseri G. Innovations in Computer Science and Engineering: Proceedings of 7th ICICSE: Springer Singapore; 2020.

Online access information for "Artificial Intelligence Unravels Interpretable Malignancy Grades of Prostate Cancer on Histology Images"

The clustered Images can be accessed and downloaded from the following links:

A) https://drive.google.com/file/d/13Rg1wyRhV4MjrxZF_rT5tj4r2QnG_rY2/view?usp=sharing
B) https://drive.google.com/file/d/1HgcojqowM3PO9vedMcbiJRjJ8AiwCTYi/view?usp=sharing
C) https://drive.google.com/file/d/1dIcNUfM02Qq5ejLonrU3A_dpijEGtH7i/view?usp=sharing
D) https://drive.google.com/file/d/1hetmgaXd1PkOdGHUM_EVQRrLSrG-ZK-n/view?usp=sharing

Specific viewer tools (e.g., QuPath) may be required to open these images.
